# Interaction of outer membrane vesicles of the oral pathogen *Porphyromonas gingivalis* with human red blood cells disclosed by proteome analyses

**DOI:** 10.1016/j.crmicr.2026.100674

**Published:** 2026-10-30

**Authors:** Marines du Teil Espina, Rosario del Carmen Flores-Vallejo, Claudia Hirschfeld, Stefano Grasso, Pim Rozendal, Anke Trautwein-Schult, Dörte Becher, Giorgio Gabarrini, Jan Maarten van Dijl

**Affiliations:** a University of Groningen, Department of Medical Microbiology, University Medical Center Groningen, 9713 GZ, Groningen, the Netherlands; b Wageningen Bioveterinary Research, 8221 RA, Lelystad, the Netherlands; c European Institute of Innovation and Technology (EIT), Healthy Ageing PhD School. Campus University Medical Center Groningen, University of Groningen, 9713 GZ, Groningen, the Netherlands; d University of Greifswald, Department of Microbial Physiology and Molecular Biology, Institute of Microbiology, Greifswald, 17489, Germany; e University of Greifswald, Department of Microbial Proteomics, Institute of Microbiology, Greifswald, 17489, Germany; f Lesaffre International, 101 Rue de Menin, Marcq-en-Barœul 59700, Marquette-lez-Lille, France; g ImmunoIVD, Nacka Strand, Stockholm, 13152, Sweden

**Keywords:** *Porphyromonas gingivalis*, OMVs, Proteome, PPAD, T9SS, Erythrocytes, Hemolysis, Hemagglutination

## Abstract

*Porphyromonas gingivalis* is a keystone oral pathogen implicated in periodontitis and several other pro-inflammatory or degenerative diseases. Previous studies showed that outer membrane vesicles (OMVs) produced by this Gram-negative bacterium are loaded with virulence factors, including the *P. gingivalis* peptidyl-arginine deiminase (PPAD). However, our understanding of strain-specific OMV features and the impact of PPAD on OMV cargo composition was so far limited. Therefore, the present study was aimed at comparing OMV proteomes of six different *P. gingivalis* strains, including PPAD-proficient and -deficient variants of the type strains W83 and ATCC 33277, and two clinical isolates. Per strain, 100–203 OMV-borne proteins were identified, originating mostly from the bacterial periplasm and outer membrane. The OMV core proteome included 79 orthogroups representing 11 biological pathways. Interestingly, 23 identified OMV cargo proteins were related to the type IX secretion system (T9SS) for export of virulence factors, invariably including all four components of the so-called T9SS attachment complex. PPAD-deficiency altered the OMV protein content by ∼18% compared to OMVs from the respective PPAD-proficient strains. Lastly, the investigated OMVs invariably carried multiple proteins involved in iron and heme acquisition. Accordingly, we demonstrate for the first time that *P. gingivalis* OMVs are highly potent inducers of hemagglutination and to lesser extent hemolysis of human erythrocytes. In conclusion, our present proteome analyses disclose interactions of *P. gingivalis* OMVs with human erythrocytes related to heme and iron acquisition, and they suggest that attachment to erythrocytes could represent a plausible mechanism for OMV dissemination throughout the human body.

## Highlights

• First definition of the *P. gingivalis* outer membrane vesicle (OMV) core proteome.
• The presence of Type IX secretion system components is a conserved OMV feature.
• The peptidyl-arginine deiminase PPAD impacts the OMV cargo composition.
• OMVs of *P. gingivalis* have various potential roles in heme and iron acquisition.
• *P. gingivalis* OMVs trigger hemagglutination and hemolysis of human erythrocytes.

## Graphical abstract

**Figure:**
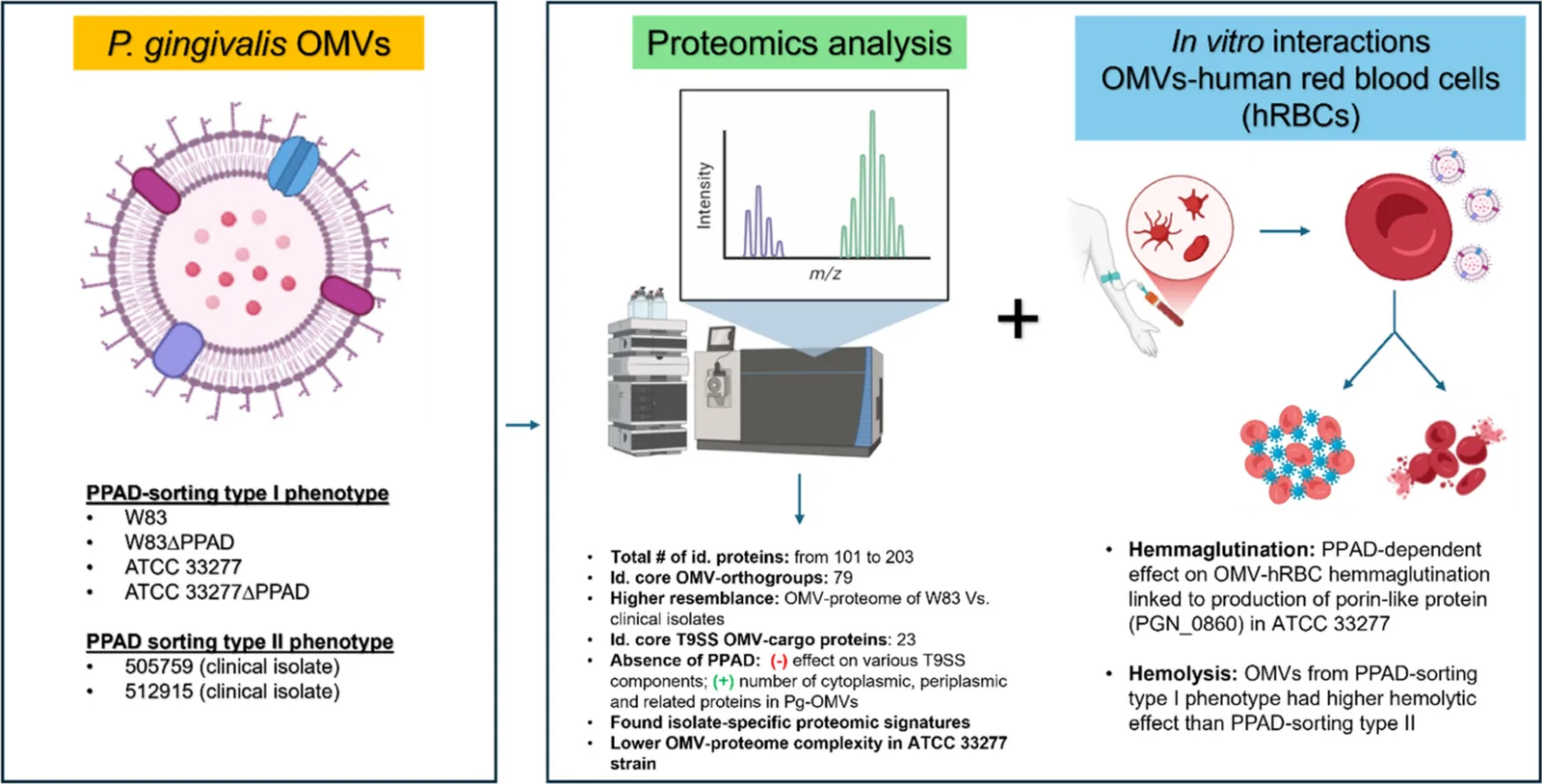

## 1 Introduction

*Porphyromonas gingivalis* is a keystone oral pathogen that drives the development of periodontitis (Hajishengallis et al., 2012). In addition, this Gram-negative bacterium has been implicated in a variety of other pro-inflammatory and degenerative diseases, such as rheumatoid arthritis and Alzheimer’s disease (Dominy et al., 2019; du Teil Espina et al., 2019). The pathogenicity of *P. gingivalis* depends on a diverse arsenal of virulence factors that are exported from the cytoplasm and, subsequently, either retained in the bacterial outer membrane, secreted in a soluble form, or sorted into outer membrane vesicles (OMVs) that are released into the extracellular milieu of the bacterium (Veith et al., 2014; Lasica et al., 2017; Gabarrini et al., 2018a).

The OMVs of *P. gingivalis* are membrane-enclosed spherical nanostructures with an average diameter of ∼120 nm (Xu et al., 2026). They are produced through outer membrane blebbing, and possess a cargo enriched in proteinaceous virulence factors (Ellis and Kuehn, 2010; Veith et al., 2014; Wang et al., 2025). Among these virulence factors are potent cysteine proteases, which are known as the gingipains RgpA/RgpB and Kgp that cleave proteins at arginine or lysine residues, respectively (Mahmud et al., 2023). These gingipains are crucial for the acquisition of nutrients such as heme, host colonization and the subversion of human immune defenses (Popadiak et al., 2007; du Teil Espina et al., 2022a Liu et al., 2024; Widziolek et al., 2025). In addition, OMVs harbor a citrullinating enzyme known as the *P. gingivalis* peptidyl-arginine deiminase (PPAD), which catalyzes the conversion of peptidyl-arginine into peptidyl-citrulline (Gabarrini et al., 2015, 2018a, 2018b). Through this activity, PPAD contributes to TLR2 activation, evasion of phagocytosis by neutrophils and macrophages, bacterial aggregation and a decreased OMV charge (Stobernack et al., 2018; Vermilyea et al., 2021; Wielento et al., 2022; du Teil Espina et al., 2022a, 2022b; Xu et al., 2026). Furthermore, PPAD is required for optimal activity of RgpA and RgpB, and it limits (self)cleavage of these arginine-specific proteases (Wegner et al., 2010; Maekawa et al., 2014; Stobernack et al., 2016, 2018; Xu et al., 2026). Lastly, PPAD-dependent citrullination has garnered general interest, as it may represent a mechanistic link between periodontitis and rheumatoid arthritis, which is characterized by anti-citrullinated protein antibodies (Wegner et al., 2010; Sokolove et al., 2014; Hecht et al., 2015; Stobernack et al., 2018; Alghamdi et al., 2019; du Teil Espina et al., 2019; Krutyhołowa et al. 2022).

Gingipains, PPAD and other virulence factors of *P. gingivalis* are localized to the bacterial outer membrane or the extracellular milieu *via* the type IX secretion system (T9SS), which is also known as the Por secretion system (PorSS). This secretion system is specific to certain species of the *Bacteroidetes* phylum (Sato et al., 2010, 2013; Gorasia et al., 2015; Lasica et al., 2017; Veith et al., 2017; Gorasia et al., 2020a, 2020b). Upon T9SS-facilitated export, proteins will remain attached to the outer membrane *via* a covalent A-lipopolysaccharide (LPS) modification, and they collectively form an electron-dense surface layer that is maintained in the OMVs upon blebbing (Lasica et al., 2017; Veith et al., 2017; Gorasia et al., 2020a). In a previous study, we analyzed the growth media of 93 clinical *P. gingivalis* isolates and observed that, in most cases, PPAD is detectable both in soluble and OMV-bound states (Gabarrini et al., 2018a). However, 11 clinical isolates displayed drastically reduced levels of outer membrane- and OMV-bound PPAD. This phenotype is associated with a Q373K amino acid substitution in PPAD, which hinders the A-LPS modification (Gabarrini et al., 2018a, 2018c). The two types of isolates that predominantly produce either A-LPS-modified PPAD or unmodified PPAD are, respectively, referred to as the PPAD sorting types I and II (Gabarrini et al., 2018a).

During the past decade, OMVs of *P. gingivalis* have been implicated in nutrient acquisition, biofilm formation and survival during stress (Cecil et al., 2019; Li et al., 2026; Xu et al., 2026). More importantly from a biomedical perspective, *P. gingivalis* strains sort major virulence factors into their OMVs, thereby turning them into specialized delivery vehicles that can target cells of the innate and adaptive immune systems (Gui et al., 2016; Olsen et al., 2016; Okamura et al., 2021; du Teil Espina, 2022a; Polishchuk et al., 2025; Wu et al., 2025; Xu et al., 2026). Furthermore, blood-borne OMVs may reach distant sites in the human body including the brain, where they can even cross the blood-brain barrier (BBB) (Dominy et al., 2019; Han et al., 2019; Jones et al., 2020; Gong et al., 2022; Pritchard et al., 2022 Yoshida et al., 2023; Qiu et al., 2025; Xu et al., 2026). In this context, it is important to consider potential OMV interactions with erythrocytes (here referred to as red blood cells [RBCs]), especially since RBCs represent the most abundant cell type in the human bloodstream (D’Alessandro et al., 2017). Notably, RBCs have the intrinsic capacity to migrate extensively through the human body, and some RBC components even reach across the BBB in the form of RBC-derived extracellular vesicles (Yang et al., 2024). Since it has been shown that *P. gingivalis* bacteria can hijack RBCs through various mechanisms (Belstrøm et al., 2011; Damgaard et al., 2016; Liu et al., 2024), it is well conceivable that RBCs serve as transport vehicles for *P. gingivalis* OMVs. To the best of our knowledge, only one study has thus far addressed the possible effects of *P. gingivalis* OMVs on RBCs from sheep, showing that binding and aggregation of RBCs can happen via the OMV-associated fimbriae-like lipoprotein PG1881 (Rothenberger et al., 2024).

To understand the full spectrum of possible OMV functions, there is a need to further characterize the proteome cargo of *P. gingivalis* OMVs with a focus on possible strain-specific variations. This should also include OMV proteomes from clinical isolates for which biodata is lacking as most studies were, thus far, primarily focused on OMVs from reference strains (Veith et al., 2014; Mantri et al., 2015; Lasica et al., 2017; Veith et al., 2018; Larsen et al., 2020, 2021). Based on these considerations, the aims of our present study were: (i) to perform a proteome-wide characterization of OMV-borne proteins from *P. gingivalis* type strains and clinical isolates belonging to the PPAD sorting types I and II; and (ii) to compare the OMV proteomes of PPAD-proficient and PPAD-deficient strains. Since these analyses identified OMV-borne proteins involved in hemagglutination and hemolysis, an additional aim was (iii) to investigate the interactions of OMVs from the different investigated strains with human RBCs *in vitro* with a focus on hemagglutination and hemolysis.

## 2 Materials and methods

### 2.1 Bacterial culture and OMV preparation

#### 2.1.1 Bacterial strains and cultures

For the present study, we applied the *P. gingivalis* type strains W83 (Roeterink et al., 1984) and ATCC 33277, as well as the respective PPAD-deletion mutants (W83ΔPPAD and ATCCΔPPAD) (Wegner et al., 2010; Gawron et al., 2014). Additionally, we used the clinical strains 505759 and 512915, which represent the PPAD sorting type II (Gabarrini et al., 2018a). All strains were grown on Blood Agar Base No.2 plates (BA2) supplemented with 5% v/v sheep blood, 0.5 mg/L hemin, and 1 mg/L menadione for 5 days at 37 °C under anaerobic conditions. Phenotypical characteristics were visualized for each *P. gingivalis* strain used. Special attention was given to the hemolysis patterns on the BA2 agar plates, which were documented after 15 days of incubation.

To prepare liquid cultures, approximately half of the colonies on a plate were used to inoculate 30 mL of culture medium, consisting of brain heart infusion (BHI) broth supplemented with 0.05% (w/v) l-cysteine, 0.5 mg/L hemin and 1 mg/L menadione. For OMV isolations, a minimum of 60 mL of culture was prepared for each strain. *P. gingivalis* liquid cultures were grown anaerobically for ∼24–26 h until an optical density at 600 nm (OD_600_) of ∼2.5 to ∼3.5 was reached (**Supplementary Figure S1, A**).

#### 2.1.2 OMV isolation, purification, and quantification

Liquid cultures of each *P. gingivalis* strain were centrifuged in 50 mL tubes at 8000 x *g* for 15 min at 4 °C to remove the bacterial cells. The supernatant was immediately and carefully removed and stored in a separate tube. Subsequently, the supernatant was filtered through a 0.22 µm filter, to remove any potential contamination by bacterial cells. The cell-free supernatant was then ultracentrifuged at 100,000 x *g* for 18 h at 4 °C in an Optima™ XL-80 K ultracentrifuge using a SW32-TI rotor (Beckman Coulter). The resulting pellet was washed with 4.0 mL of phosphate-buffered saline (PBS) and subjected to ultracentrifugation at 100,000 x *g* for 2 h at 4 °C in an Optima™ Max-XP ultracentrifuge (Beckman Coulter) using an MLA-80 rotor, which was also used for all the following ultracentrifugation steps.

To purify OMVs, iodixanol (Optiprep™) density gradient centrifugation was performed. To this end, the OMV pellet was resuspended in 500 µL of a 50% (w/v) Optiprep solution in 0.85% (w/v) NaCl, 60 mM HEPES-NaOH (pH 7.4; buffer A). An iodixanol gradient was subsequently created by sequentially layering decreasing concentrations of iodixanol on top of this 50% layer. Specifically, 900 µL of each 40%, 30%, 20%, and 10% iodixanol solutions, prepared in 0.85% (w/v) NaCl 10 mM HEPES-NaOH (pH 7.4; buffer B), were very carefully overlaid in this order from bottom to top. After layering the solutions, the tubes were ultracentrifuged for 2 h at 100,000 x *g* at 4 °C. During this time, the OMVs migrated to fractions with iodixanol densities of <50%, resulting in a clearly visible OMV-containing colored zone (**Supplementary Figure S1, B**). To determine exactly in which fractions the OMVs were located, 300 µL aliquots were collected from the top of the gradient. From each fraction, a 50 µL aliquot was used for protein precipitation with 10% trichloroacetic acid (TCA) as previously described (Gabarrini et al., 2018a, 2018b). The resulting protein pellet was then resuspended in 40 µL of gel loading buffer, consisting of lithium dodecyl sulphate (LDS) sample buffer 4x (Life Technologies) and sample reducing agent 10x (Life Technologies) in MilliQ water, and LDS-PAGE was performed as described in the Methods Section 2.2.8 below. For each sample, SimplyBlue Safe-Staining and Western-blotting with specific polyclonal rabbit antibodies against the OMV and outer membrane marker protein 41 (Omp41) was performed (see **Methods** Section 2.2.8; Gabarrini et al., 2018a) (**Supplementary Figure S1, C-D**). The *P. gingivalis* OMVs were concentrated in the 20–30% iodixanol fractions (**Supplementary Figure S1, C-D**) as previously shown (Cecil et al., 2016). Lastly, the OMV-containing fractions were combined, and buffer B was added to the maximum volume of the ultracentrifuge tube (4 mL). Overnight ultracentrifugation was performed (100,000 x *g* at 4 °C), the pelleted OMVs were washed with PBS, and the OMVs were recollected by ultracentrifugation for 2 h using the same conditions. OMVs were then resuspended in PBS and aliquots were stored at −80 °C. To determine the protein concentration of OMV samples, the Pierce™ bicinchoninic acid (BCA) Protein Assay Kit (Thermo Fisher Scientific) was used according to the manufacturer’s instructions. Of note, OMVs were first treated with 2% sodium dodecyl sulfate (SDS) before performing the protein quantification, as previously recommended (Morton and Evans, 1992).

#### 2.1.3 Biophysical characterization of OMV properties

The diameter (nm), zeta potential (mV) and particle concentration (particles/mL) of purified OMVs were determined using purified OMVs from 3 independent, biological replicate bacterial cultures. OMVs were filter-sterilized using a 0.22 µM polyvinylidene fluoride (PVDF) membrane (Avantor. PE, USA). Measurements were carried out using a ZetaView® PMX-120 Nanoparticle Tracking Analysis (NTA) instrument (Particle Metrix GmbH, Ammersee, Germany) equipped with a Z-NTA cell assembly, blue laser (laser wavelength 488 nm, 40 mW), and a Complementary Metal-Oxide Semiconductor (CMOS) camera with 640 × 480-pixel resolution. Stock solutions were prepared to a normalized concentration of 100 µg/mL of total protein content and diluted with Dulbecco's phosphate-buffered saline buffer (DPBS, pH 7.0) with a final dilution factor between 1:1 × 10^5^ to 1:3 × 10^5^ to meet the particle saturation parameters of the NTA equipment. Videos in NTA mode were recorded at 11 positions across the measurement chamber in 5 s increments at a framerate of 30 Frames Per Second (FPS) with a camera shutter speed at 100 s^−1^ and sensitivity at 80. Temperature was controlled at 25 °C during the analysis. Videos were processed with the ZetaView software v8.05.16 SP3. Particles between 10 and 1000 nm in diameter with a minimum trace length of 15 frames and a minimum brightness of 30 were included in the analysis. Data was reported as the mean value of each parameter ± standard deviation.

### 2.2 Comparative proteomics analyses

#### 2.2.1 Sample preparation for protein identification and label-free quantification using gel lc-ms/ms

For proteome analyses by mass spectrometry (MS) a label-free quantification strategy was applied, where purified and enriched OMV protein samples from three biological replicate cultures of each strain were pre-fractionated by LDS-PAGE. To this end, OMV aliquots of 30 µg protein were loaded onto the gel, which was run for 5 min at 200 V. Upon overnight SimplyBlue SafeStaining, the different gel lanes (5–10 mm in length) were excised and cut into smaller pieces per sample (Bonn et al., 2014). After removal of salt and non-proteinaceous contaminants by incubation in a buffer containing 0.2 M ammonium bicarbonate in 30% (v/v) acetonitrile, the gel-pieces were dried in a vacuum centrifuge and rehydrated with an aqueous solution of trypsin (2 ng/µl). Proteins were then digested with trypsin overnight, and the resulting peptides were eluted in ultrapure water and analyzed by MS.

#### 2.2.2 LC-MS/MS analyses

All proteomic samples were analyzed by reversed-phase liquid chromatography (LC) electrospray ionization MS/MS using an LTQ Orbitrap Velos Elite (Thermo Fisher Scientific) as previously described (Stobernack et al., 2018). In brief, in-house self-packed nano-LC columns (100 µm x 20 cm) containing reversed-phase C18 material (3 µm, ReproSil-Pur 120-AQ, Dr. Maisch GmbH) were used to perform LC with an Easy-nLC1200 system (Thermo Fisher Scientific). The peptides were loaded with solvent A (0.1% [v/v] acetic acid). Subsequently, the peptides were eluted by a non-linear binary gradient of 86 min from 2% to 99% solvent B (0.1% [v/v] acetic acid, 94.9% [v/v] acetonitrile) in solvent A at a constant flow rate of 300 nL/min.

After sample injection into the mass spectrometer, a full scan was recorded in the Orbitrap (*m/z* 300 - 1700) with a resolution of 60,000 at 400 *m/z*. The 20 most abundant precursor ions were consecutively isolated in the linear ion trap and fragmented via collision-induced dissociation. Unassigned charge states as well as singly charged ions were rejected and the lock mass correction was enabled. Selected ions were fragmented with 35% normalized collision energy and analyzed by data-dependent MS/MS in the linear trap quadrupole.

#### 2.2.3 Database for protein identification

For protein identification, spectra were searched separately using MaxQuant with the implemented Andromeda search algorithm (version 1.6.17.0) (Cox and Mann, 2008; Cox et al., 2011) against specific databases for each *P. gingivalis* strain. For the W83 type strain, the proteome UP000000588 contained 1926 entries and for ATCC 33277, the proteome UP000008842 contained 2024 entries. These proteomes were downloaded from Uniprot (www.uniprot.org) on 19/03/2020 (The UniProt Consortium, 2021). Furthermore, the proteome of the clinical isolate 512915 contained 2214 entries and the one of the clinical isolate 505759 contained 2192 entries. Of note, the identifiers for 512915 and 505759 are in RAST format, whereas for the ATCC 33277 and W83 strains, Uniprot IDs and connected gene names were available.

#### 2.2.4 Proteome annotation

Since the previously annotated proteomes appeared to lack certain proteins, BLAST searches were performed using the RefSeq NCBI entries NC_010729.1 1 and NC_002950.2, for the ATCC 33277 and W83 strains, respectively. Starting with the well-annotated genome sequence of the *P. gingivalis* ATCC 33277 strain (Gabarrini et al., 2020), each protein of this type strain was used to search both the genomes and proteomes of the three other strains used in this study by the tblastn and blastp algorithms, respectively (BLAST+ v. 2.8.1) (Camacho et al., 2009). Query proteins with the following characteristics were identified as missing: tblastn alignment length > 50 AA, tblastn percentage of identical matches > 50%, blastp e-value > 0.001. These proteins could not be retrieved by blast analyses of proteome against proteome, but they were nonetheless identified with a high degree of confidence as being encoded by the different genome sequences. Following this procedure, we identified and added 62 additional proteins for strain 505759, 42 for strain 512915, and 63 for the W83 strain. Furthermore, 41 additional proteins matching the described parameters were identified for the ATCC 33277 strain using the 512915 strain as a reference. Of these 41 proteins, the only two non-hypothetical proteins were added to the ATCC 33277 proteome.

#### 2.2.5 Protein identification and label-free quantification

For protein identification, spectra were searched separately against the corresponding *P. gingivalis* strain-specific databases using MaxQuant with the implemented Andromeda search algorithm (version 1.6.17.0, Max Planck Institute of Biochemistry) (Cox and Mann, 2008; Cox et al., 2011). Parameters for precursor mass deviation were set to 4.5 ppm and 0.5 Da for fragment mass tolerance. A false discovery rate (FDR) of 0.01 on protein, peptide, and spectral levels was applied, as well as a minimum peptide length of seven amino acids and two unique peptides per protein. Full tryptic specificity with a maximum of two missed cleavage sites was applied. Variable modifications were oxidation on methionine (M) and N-terminal acetylation (Protein N-term), citrullination on arginine (R) and deamidation on asparagine and glutamine (NQ). The label-free quantification option LFQ was activated (min. ratio count set to two) as well as the option “match between runs” for the bioreplicates of each strain. Furthermore, MaxQuant’s generic contamination list was included and MaxQuants’ normalization option was enabled as well.

For shotgun proteomic data analyses the software Perseus v.1.6.14.0 was used (Tyanova et al., 2016). The data from MaxQuant output files were filtered for contaminations, reversed, and only identified by site hits. The quantification of comparative proteome analyses for the *P. gingivalis* wild-type strains and ∆PPAD mutants was based on MaxQuant’s LFQ (Cox et al., 2014). For protein identification, data were filtered for at least two unique peptides per protein in three out of three bioreplicates. Proteins were considered for quantification if an LFQ value was present in all three biological replicates of each strain. Statistical evaluation of quantified proteins was performed by applying a two‑sided Student’s t‑test. Proteins were considered differentially expressed when they exhibited a log₂ fold change with an absolute value ≥ 0.58 (corresponding to a ≥ 1.5‑fold change in abundance) and a p‑value ≤ 0.05.

#### 2.2.6 Validation of potential citrullination sites

Since classical database search algorithms are known to overestimate the number of potentially citrullinated peptides and citrullination sites, a manual validation of all spectra including potential citrullination was performed as previously described (Lee et al., 2018). In brief, spectra were filtered for posterior error probability (PEP score) < 0.01, Andromeda score >100 and Isotope index <1. Moreover, the spectra were inspected for the presence of diagnostic ions with the neutral loss of isocyanic acid, the presence of the ammonium ion at 130.097 *m/z* and incomplete fragment ion series without N/Q ambiguity.

#### 2.2.7 Bioinformatic analyses

For comparisons between OMV proteomes obtained with different databases, we first created orthogroups of the proteomes of all investigated strains using the OrthoFinder v.2.3.11 methodology (Emms and Kelly, 2015, 2019), which infers orthogroups of protein-encoding genes with great accuracy. An orthogroup is defined as the set of genes from different species that descend from a single gene in the last common ancestor. Subsequently, the presence of each orthogroup to which the identified proteins belong was determined for all the strains analyzed. Orthogroups of particular importance for this study, such as hemagglutinins and gingipains, were manually curated. For this purpose, the standalone tool blastp from the suite BLAST+ v. 2.9.0 (Camacho et al., 2009) was used to pair orthologous proteins within an orthogroup. Additionally, due to the high similarity between hemagglutinins and gingipains, ClustalO was used to further confirm the manual curation (Sievers et al., 2011). Prediction of subcellular protein localization was performed as described previously (Gabarrini et al., 2020). In addition, the predicted subcellular localization of putative and known T9SS cargo proteins was manually curated based on previous literature. Visualization of the number of proteins assigned to each localization was performed using GraphPad Prism v. 8.0.1. The GO terms associated with particular protein domains, analyzed with InterProScan v. 5.27 against InterPro v. 66.0 (Jones et al., 2014; Blum et al., 2021), were used for REVIGO analyses to determine the biological pathways in which MS-identified OMV proteins of each strain are involved. Lastly, Venn diagrams were created using the web-based tool “InteractiVenn” (Heberle et al., 2015).

#### 2.2.8 LDS-PAGE and western blotting

For LDS-PAGE, OMV samples prepared as described above were incubated for 10 min at 95 °C and separated on 10% NuPAGE gels (Invitrogen). Subsequently, the gels were either stained with SimplyBlue SafeStain (Thermo Fisher Scientific) or transferred from the gel to a 0.45 μm nitrocellulose membrane (Whatman). Membranes with bound proteins were blocked by overnight incubation with 5% skim milk in PBS at 4 °C. After washing once with PBS-Tween20 (PBS-T), the membranes were incubated for 2 h at room temperature with specific polyclonal rabbit antibodies diluted 1:10,000 in PBS-T. Specifically, the applied antibodies were raised against Omp41, PPAD, RgpA/B or Kgp (Gabarrini et al., 2018a; Xu et al., 2026). Subsequently, the membrane was washed 5 times with PBS-T prior to incubation for 1 h at room temperature with a secondary IRDye 800CW-labeled goat anti-rabbit antibody (LI-COR Biosciences) diluted 1: 10,000 in PBS-T. After five PBS-T washes to remove any unbound secondary antibody, the membrane was washed twice with PBS and analyzed with the LI-COR Odyssey infrared imaging system (LI-COR Biosciences) or a Typhoon Biomolecular Imager (Amersham).

### 2.3 Interaction of OMVs with human red blood cells

#### 2.3.1 Hemagglutination assay with *P. gingivalis* OMVs

Human red blood cells (hRBCs, A type) were harvested from approximately 10 mL of blood of a healthy donor, collected in K_2_-EDTA-coated vacutainer tubes, and separated with the Lymphoprep® gradient (Stem cell technologies, Vancouver, Canada), considering methods of previous reports with modifications (Evans et al., 2013; Stobernack et al., 2018). In particular, the fraction that corresponded to hRBCs and neutrophils was washed with 1 mL of 150 mM NaCl followed by centrifugation for 5 min at 500 x *g* and 20 °C. Stock solutions of hRBCs (2% v/v) were prepared in a modified version of the Saline Adenine Glucose Mannitol (mSAGM) storage medium (Zehnder et al., 2008) in Dulbecco's phosphate-buffered saline (DPBS, final pH 6.1), and stored for a maximum of 4 weeks at 4 °C. The hRBCs were allowed to precipitate in the storage medium for 48 h prior usage for subsequent bioassays. On the day of the experiment, hRBC stock solutions were washed three times with DPBS (pH 7.2) by centrifugation at 500 x *g* (5 min, 20 °C) to remove the mSAGM medium and lysed cells, and to retain only intact hRBCs. After washing, 50 µL aliquots of the 2% (v/v) intact-hRBC suspension were loaded into the wells of a U-shaped 96-well polypropylene microplate without-coating (Greiner®) containing the same volume of double serial dilutions of OMV stocks in DPBS (in-test concentrations 2.5–0.005 µg/mL). The plate was then incubated at 37° C for one hour. Lectin (PHA-P, Sigma Aldrich ®) in DPBS was used as a positive control (in-test concentrations from 500 to 0.98 µg/mL) for hemagglutination, and DPBS was used as a negative control. After the incubation, the hemagglutination titer (HaT) was qualitatively determined by visual macroscopic inspection. It was defined as the minimum concentration of total protein content of OMVs at which the hRBCs completely agglutinated at the bottom of the plate. Each treatment was tested with two technical replicates in two independent experiments.

#### 2.3.2 Hemolysis assay with *P. gingivalis* OMVs

A hemolysis assay was conducted with hRBCs by adapting a previously described methodology with modifications to evaluate the hemolytic effect of *P. gingivalis* OMVs (He et al., 2020). First, the hRBC stock was washed with DBPS as described above for the hemagglutination assays. Subsequently, a volume of 50 µL containing 2 × 10^5^ intact-hRBCs in DPBS was loaded into each well of a U-shaped 96-well polypropylene microplate without coating (Greiner®) containing an equal volume of double serial dilutions of the OMVs also in DPBS (final in-test concentrations from 25 to 3.25 µg/mL). The plate was then incubated at 37 °C for 48 h and centrifuged at 500 x *g* for 5 min. Subsequently, 20 µL of the supernatant from the OMV-treated hRBCs was transferred into a flat-bottomed 96 well plate containing 180 µL of DPBS. To quantify hemolysis the plate was read at an optical density of 410 nm (OD_410_) in a Biotek Epoch2® plate reader. DPBS was used as the negative control for hemolysis, while 1% (w/v) SDS was used as positive control (100% hemolysis). Each treatment was tested with two technical replicates in two independent experiments. Subsequently, the hemolytic activity was quantified as the percentage of hemolysis in accordance with the Standard Practice for Assessment of Hemolytic Properties of Materials as issued by the American Society for Testing and Materials (ASTM F-756-00). To this end, we applied a previously reported equation (ASTM, 2020), which was adapted to our assay conditions as: 100 x (OD_410_ OMV-treated hRBCs - OD_410_ negative control)/(OD_410_ of positive control - OD_410_ negative control). The results were then reported as the arithmetic mean of the percentage of hemolysis ± standard deviation obtained from each treatment (n = 48). Additionally, the concentration of OMVs capable of inducing a hemolytic effect in 50% of the hRBCs (HEC_50_) per strain of *P. gingivalis* was determined through linear regression analysis from 4-point regression plots obtained in Graphpad Prism® v.8.0.1.

#### 2.3.3 Biostatistical analyses

The results from the hemolysis assay (n = 48) were analyzed in JMP® v. 16.0.0. through a linear regression model based on the least squares method (LR-LSM). A histogram of the distribution of the residuals was plotted and the goodness of the fit of the model was verified through the Shapiro-Wilkins and the Anderson-Darling tests followed by an analysis of variance (ANOVA) (significance α=0.05). The independent experimental factors considered for this analysis were: (1) the type of OMV-producing *P. gingivalis* strain; (2) the OMV concentrations (25–3.25 µg/mL); and (3) each independent experiment. A p-value ≤ 0.05 was considered to indicate statistical significance. The differences between the independent experimental factors were analyzed through a Pareto plot. Finally, a Tukey’s post hoc test was carried out to evaluate whether there were differences in the hemolytic activities of OMVs from the different bacterial strain.

#### 2.3.4 Medical ethics committee approval

Blood donations from healthy volunteers were collected based on written informed consent with approval of the medical ethics committee of the University Medical Center Groningen (UMCG; approval no. Metc2012-375), and in accordance with the Declaration of Helsinki guidelines.

## 3 Results and discussion

### 3.1 General characterization of *P. gingivalis* OMV proteomes

#### 3.1.1 OMV characterization, identification of OMV-associated proteins, and comparison against reference databases

To obtain an inventory of the proteins associated with OMVs of *P. gingivalis* and the respective strain-specific variations, we analyzed the OMV proteomes of the two well-studied *P. gingivalis* type strains W83 and ATCC 33277, their respective PPAD-deficient mutants, and the two clinical isolates 505759 and 512915. The two clinical *P. gingivalis* isolates belong to the PPAD sorting type II, which was previously defined as having a PPAD mutation that hinders the binding of this protein to the outer membrane (Gabarrini et al., 2018a).

To assess the quality of purified *P. gingivalis* OMVs for proteome analyses, we determined their physicochemical properties (i.e. size, zeta potential and concentration), and we performed an NTA imaging analysis. The results obtained for the OMVs from *P. gingivalis* strains ATCC 33277 and ATCC∆PPAD, and the clinical isolates 505759 and 512915 are presented in **Supplementary Table S1, Supplementary Figure S2** and **Supplementary Videos S1-S4**. Furthermore, we documented the respective results for OMVs from the W83 and W83∆PPAD strains in a recent publication (Xu et al., 2026). In general, the size of OMVs from the ATCC 33277 and ATCC∆PPAD strains, and the clinical isolates 505759 and 512915 ranged between 100.85 nm to 120.67 nm, with zeta potentials ranging between −47.07 mV to −38.58 mV. The total concentration of particles in OMV stocks containing 100 µg/mL of total protein was between 3.70 × 10^11^ particles/mL to 4.72 × 10^12^ particles/mL. Overall, these results show that the OMV purification procedure implemented in our present study and previous studies from our group (du Teil Espina et al., 2022a, 2022b; Xu et al., 2026) allowed the effective isolation of OMVs from *P. gingivalis* that are suitable for in-depth proteomics analyses*.*

OMV-associated proteins were identified by establishing separate proteome databases for each of the investigated strains. A summary of the results of this analysis is presented in Table 1 and the details are presented in **Supplementary File S1** and **Supplementary File S2, sheet 1**. The total numbers of proteins identified in the OMVs of the *P. gingivalis* reference strains and clinical isolates ranged from 101 to 203 proteins for the ATCC 33277 and W83 reference strains. These proteins are part of 99 and 201 orthogroups, respectively. Furthermore, we identified 107 and 194 proteins in OMVs of the PPAD-deficient ATCCΔPPAD and W83ΔPPAD strains, which belong to 105 and 193 orthogroups, respectively. Lastly, 160 and 194 proteins were identified in OMVs of the clinical isolates 505759 and 512915, which belong to 159 and 193 orthogroups, respectively. Of note, more than one identified protein can be assigned to one orthogroup and, therefore, for strains ATCC 33277, ATCCΔPPAD and 505759, the number of orthogroups is slightly lower than the number of identified proteins (Table 1). Furthermore, in OMVs from most investigated strains one or two proteins could not be assigned to any known orthogroup (Table 1; **Supplementary File S2, sheet 1).** Accordingly, these unassigned proteins had to be excluded from the present cross‑strain OMV orthogroup analyses.

**Table 1:** Numbers of identified OMV proteins assigned to orthogroups.

*P. gingivalis* strain	aTotal number of identified proteins	# Identified orthogroups (# of proteins within orthogroups)	# Identified unassigned proteins
W83	203	201 (201)	2
W83ΔPPAD	194	193 (193)	1
ATCC 33277	101	99 (100)	1
ATCCΔPPAD	107	105 (106)	1
505759	160	159 (160)	0
512915	194	193 (193)	1

a For details consult Supplementary File S1 and Supplementary file S2, sheet 1.

Previous studies on the proteome of *P. gingivalis* OMVs were mostly focused on OMVs from the reference strains ATCC 33277, W83 and W50, and different numbers of identified OMV-borne proteins were reported for these strains (Veith et al., 2014; Mantri et al., 2015; Lasica et al., 2017; Veith et al., 2018; Larsen et al., 2020). For instance, in the study of Mantri et al. (2015), a total of 67 and 70 proteins were identified in OMVs from the ATCC 33277 and W83 strains, respectively. From those, only 12 proteins were unique to the ATCC 33277 strain. These included minor components of the major fimbriae, FimC, FimD, and FimE. In contrast, 23 proteins were uniquely identified in OMVs of the W83 strain, which included a PorT protein and several proteins with a tetratricopeptide repeat (TPR) domain (Mantri et al., 2015). In the study by Larsen et al. (2020), 88 OMV-associated proteins were reported for the W83 strain, and 112 for the W83ΔPPAD strain. The numbers of identified OMV-associated proteins in our present study are more similar to the numbers reported by Veith et al. in two previous studies on the OMV proteome of the *P. gingivalis* strain W50, where 151 or 180 proteins were identified (Veith et al., 2014, 2018). Interestingly, we identified ∼80–90% of the latter proteins in our present study (**Supplementary File S2, sheet 3)**. Conversely, in OMVs of the ATCC 33277 strain and its PPAD-deficient derivative, we identified 101 and 107 proteins, respectively (Table 1), and these numbers more closely resemble the numbers of OMV-associated proteins identified by Larsen et al. (2020). At present, it is not clear why the numbers of identified proteins in the reference strains of the previous and our present analyses differ, but this most likely relates to differences in OMV purification, sample preparation and/or the subsequent MS analyses (Li et al., 2026). For example, we purified the isolated OMVs using an iodixanol gradient to remove possible contaminants and to obtain concentrated OMV preparations (Klimentová and Stulík, 2015; Chutkan et al., 2012). Other causes for differences in the total numbers of identified proteins in the OMVs of *P. gingivalis* reference strains could be related to lab-to-lab strain variations or differences in culture conditions. For instance, in our study we used BHI medium supplemented with l-cysteine, hemin and menadione, whereas Mantri et al. (2015) and Larsen et al. (2020) used trypticase soy broth (TSB) supplemented with yeast extract, cysteine, hemin and menadione. Another example of variations in the media used for the analysis of OMVs produced by *P. gingivalis* is the protein-enriched version of the BHI medium, as implemented in studies of Veith et al. (2014) and Gorasia et al. (2020b) for growing the W50 strain.

#### 3.1.2 Predicted subcellular localization of identified OMV-associated proteins

The subcellular localization of proteins identified in the different *P. gingivalis* OMV preparations was predicted following a previously described pipeline (Gabarrini et al., 2020). A summary of the predicted subcellular localization of all identified proteins is presented in **Supplementary File S2, sheet 5**. As expected, the majority of proteins identified in all OMVs include predicted outer membrane and periplasmic proteins, which account for up to ∼70% of the total proteins identified in OMVs of all analyzed strains (Fig. 1). For the OMVs of most strains, ∼20% of the identified proteins were inner membrane or cytoplasmic proteins. However, the latter was not the case for OMVs of the ATCC 33277 strain and its PPAD-deficient derivative, which contained merely ∼8% of predicted inner membrane and cytoplasmic proteins. The presence of relatively large numbers of typical inner membrane and cytoplasmic proteins was also noted in previous publications on OMVs from *P. gingivalis* and other bacteria (Larsen et al., 2020; Nagakubo et al., 2020). This phenomenon might be due to the secretion of so-called ‘outer-inner membrane vesicles’ (O-IMVs) (Pérez-Cruz et al., 2015; Nagakubo et al., 2020), although a definite identification of such O-IMVs has not yet been documented for *P. gingivalis*. In addition, the presence of so-called moonlighting cytoplasmic proteins associated to OMVs of *P. gingivalis* has been reported (Veith et al., 2018). Indeed, similar to Veith et al. (2018), we identified in the OMVs of all presently analyzed *P. gingivalis* strains the cytoplasmic proteins glyceraldehyde 3-phosphate dehydrogenase (GAPDH), 4-hydroxybutyryl-CoA dehydratase and NAD-specific glutamate dehydrogenase. Interestingly, we recently observed that fimbriae from the oral pathogen *Aggregatibacter actinomycetemcomitans* contribute to maintaining bacterial cell envelope integrity, and that non-fimbriated *A. actinomycetemcomitans* strains release increased amounts of OMVs and extracellular cytoplasmic proteins (ECPs) (Fu et al., 2022, 2023, 2026). This phenomenon could possibly also explain the observed difference in cytoplasmic protein content in OMVs from the non-fimbriated *P. gingivalis* strain W83 and the fimbriated ATCC 33277 strain.

**Fig. 1:**
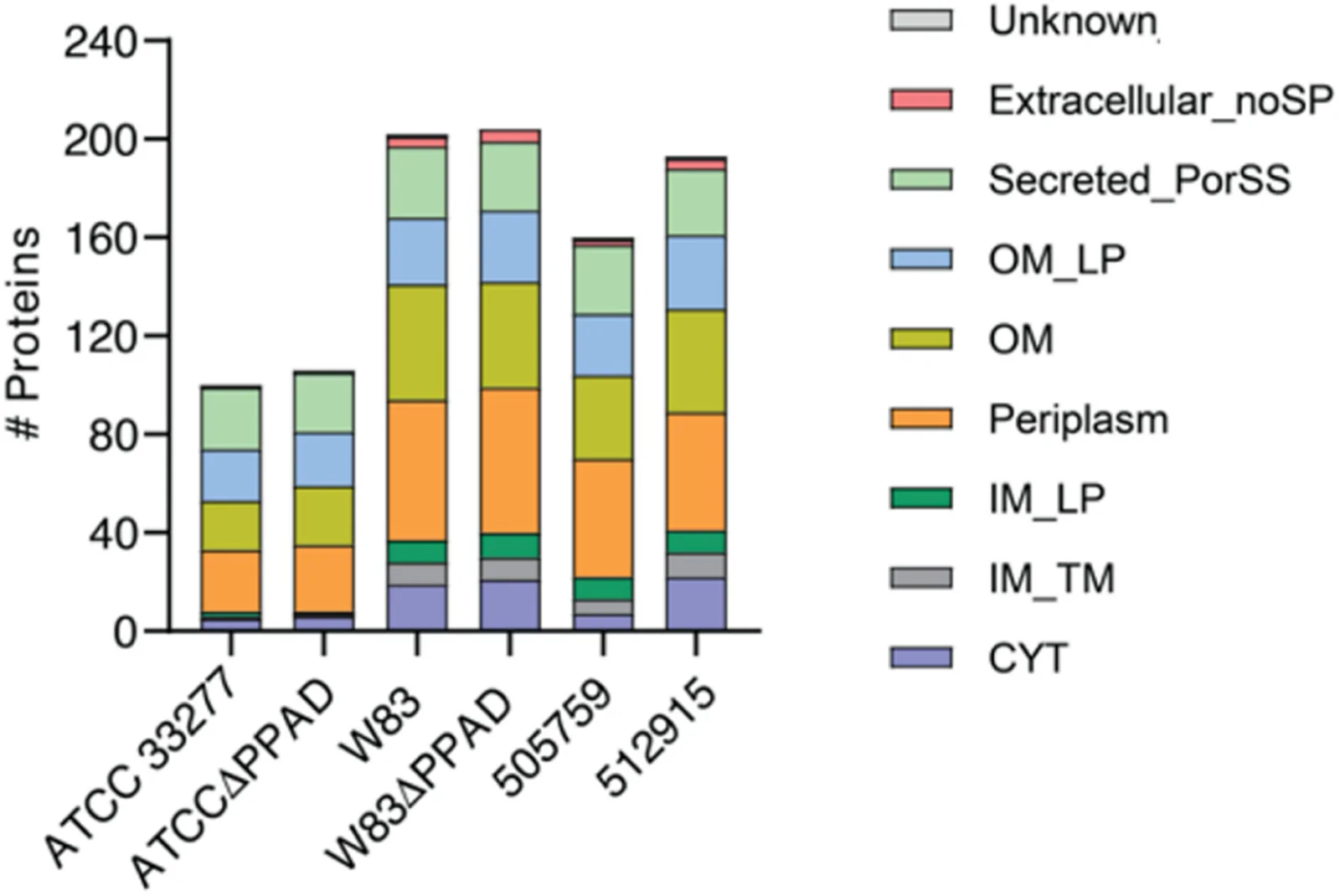
**Predicted subcellular localization of identified OMV-borne proteins per investigated *P. gingivalis* strain.** The predicted localizations of OMV-borne proteins are marked in color code. CYT, cytoplasmic; IM, inner membrane; LP, lipoprotein; noSP, not a predicted secretory protein; OM, outer membrane; PorSS, Por secretion system; SP, signal peptide; TM, transmembrane.

As observed for predicted inner membrane and cytoplasmic proteins, a relatively low number of periplasmic and outer membrane proteins was observed in OMVs of the ATCC 33277 strain and its PPAD-deficient derivative. Nonetheless, very similar numbers of T9SS-dependently secreted proteins were identified in OMVs of these two ATCC 33277 strains and OMVs of the other presently investigated strains. This suggests that the relatively lower number of identified proteins in OMVs of the ATCC 33277 strain cannot be directly explained by differences in culture conditions, OMV-sample preparation or the subsequent detection and quantification of OMV-borne proteins. Instead, this is indicative of a lower OMV-complexity in the ATCC 33277 strain compared to the other investigated strains. In particular, the relatively low number of OM-associated proteins in OMVs of the ATCC 33277 strain and their high overlap with proteins in the OMVs of the other strains suggest that apparently more virulent strains, such as W83 and the two investigated clinical isolates, shed OMVs with higher proteomic and functional complexity.

We also predicted the localization of uniquely identified OMV-associated proteins for all the *P. gingivalis* strains used in this study (**Supplementary File S2, sheet 2**). Notably, the majority of these proteins are typical inner membrane and cytoplasmic proteins. This contrasts with the observation that the majority of OMV-associated proteins are derived from the outer membrane and periplasm of *P. gingivalis* (Fig. 1).

#### 3.1.3 High relative abundance of T9SS-related OMV cargo proteins and presence of T9SS attachment complex proteins as conserved features of *P. gingivalis* OMVs

T9SS-related OMV cargo proteins were defined based on previously published datasets (Lasica et al., 2017; Veith et al., 2021), from which a curated reference list was compiled including 33 proteins (**Supplementary File S2, sheet 3**). This list was subsequently used to refine the predicted subcellular localization of identified orthogroups across all strains (marked in red in **Supplementary File S2, sheet 1**). Comparisons of the numbers of identified T9SS-related cargo proteins in OMVs from all strains investigated in our present study and the previous study by Veith et al. (2014) are presented in Fig. 2A,B. Altogether, 23 cargo proteins related to the T9SS were shared by all analyzed OMVs (Table 2), representing ∼75% of the total T9SS-related cargo proteins identified in each of the strains. The only strain that contained three uniquely identified T9SS-related proteins was W83 (Fig. 2A). Thus, the presence of T9SS-related cargo proteins is an apparently conserved feature of the OMVs of *P. gingivalis*, despite substantial differences in the overall protein composition of the respective OMVs.

**Fig. 2:**
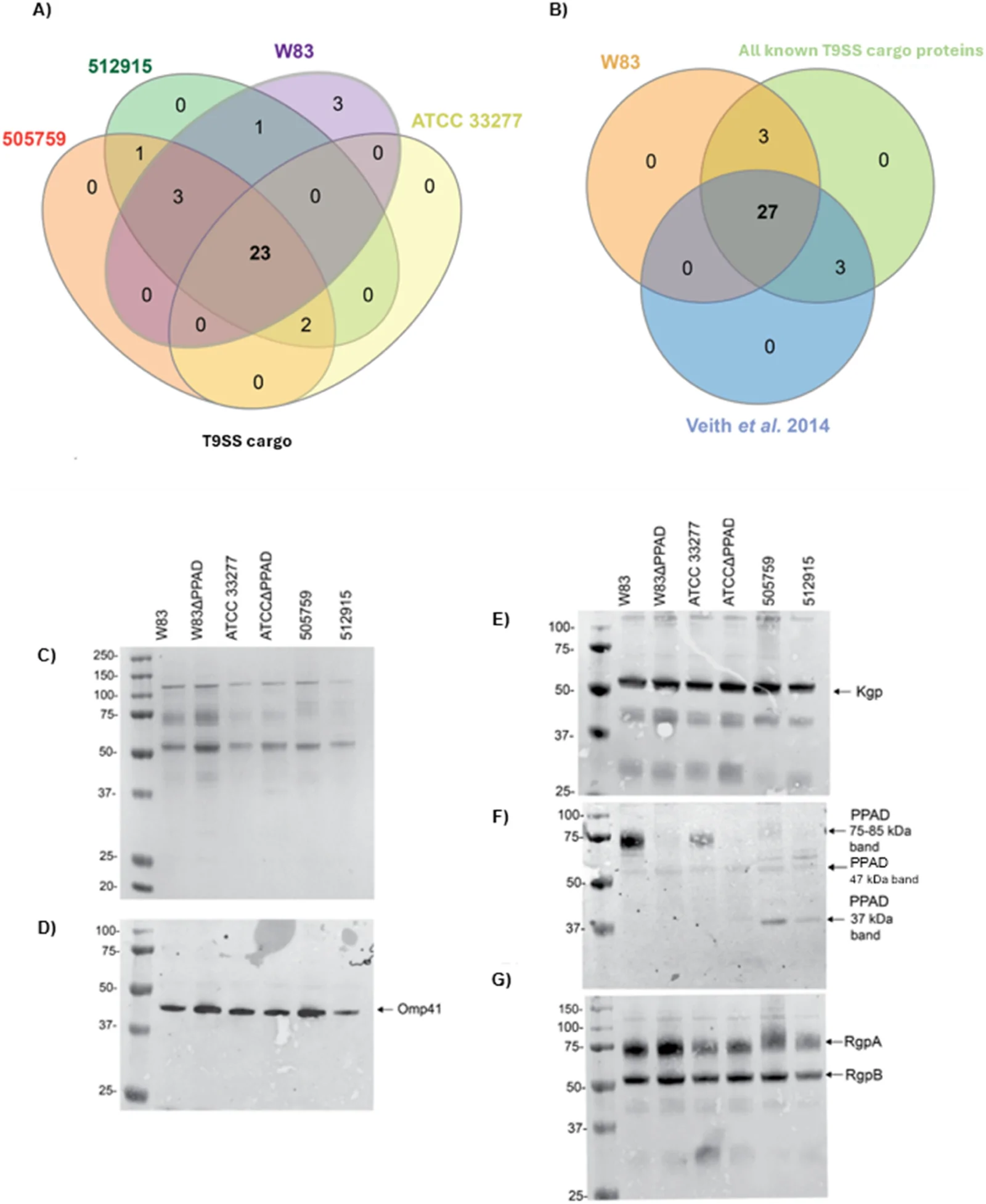
**T9SS-related cargo proteins in *P. gingivalis* OMVs identified by proteome analyses and Western blotting. (A)** Numbers of common and variant T9SS cargo proteins identified by proteomics in OMVs from *P. gingivalis* strains investigated in this study. **(B)** Numbers of common and variant T9SS cargo proteins identified by proteomics in OMVs of *P. gingivalis* strains W83 (this study) and W50 (Veith *et al*. 2014) compared to the 33 known T9SS cargo proteins. **(C)** LDS-PAGE of OMVs isolated from the different *P. gingivalis* strains investigated in this study. Proteins were visualized by SimplyBlue SafeStaining. **(D-G)** Western blotting analysis of *P. gingivalis* OMV-associated proteins with specific antibodies against: **(D)** Omp41, **(E)** KgP; **(F)** PPAD; or **(G)** RgpA/B.

**Table 2:** T9SS-related cargo proteins identified in OMVs of all investigated strains.

Gene locus tag ATCC 33277	Gene locus tag W83	Protein name
PGN_0022	PG0026	Por secretion system protein PorU
PGN_0291	PG0182	Minor fimbrium subunit Mfa5
PGN_0335	PG0232	Zinc carboxypeptidase
PGN_1611	PG0350	Internalin-related protein
PGN_1556	PG0411	Putative hemagglutinin
PGN_1466	PG0506	Gingipain RgpB *
PGN_1416	PG0553	Probable Lysyl endopeptidase PepK
PGN_0654	PG0611	Lipoprotein
PGN_0657	PG0614	Uncharacterized protein
PGN_0659	PG0616	35 kDa hemin binding protein
PGN_0693	PG0654	Por_Secre_tail domain-containing protein
PGN_1321	PG1030	Uncharacterized protein
PGN_0852	PG1374	Immunoreactive 47 kDa antigen
PGN_0898	PG1424	Peptidylarginine deiminase (PPAD)**
PGN_0900	PG1427	Periodontain (PrtT)
PGN_0509	PG1604	Immunoreactive 84 kDa antigen
PGN_1767	PG1798	Immunoreactive 46 kDa antigen
PGN_1733	PG1837	Hemagglutinin protein HagA***
PGN_1728	PG1844	Gingipain Kgp *
PGN_1970	PG2024	Gingipain RgpA*
PGN_0152	PG2102	Immunoreactive 61 kDa antigen
PGN_0123	PG2172	PorA
PGN_2080	PG2216	Uncharacterized protein

*, Marks proteins that were also detected by Western-blotting for all strains; **, marks the PPAD protein detected in OMVs of the W83 and ATCC 33277 strains and both clinical isolates, but not in PPAD-deletion mutants of the W83 and ATCC 33277 strains; ***, marks T9SS-related cargo proteins in OMVs of the investigated clinical isolates.

Interestingly, when comparing the numbers of T9SS-related cargo proteins identified in *P. gingivalis* OMVs by Veith et al. (2014, 2018) with our present identifications, we notice significant overlaps (Fig. 2B, **and Supplementary File S2, sheet 3**). Based on these combined observations, we conclude that all 33 known T9SS-related OMV cargo proteins have been identified in the presently investigated OMVs of the *P. gingivalis* reference strains and clinical isolates. However, only 23 of these proteins were commonly identified in the OMVs of all presently investigated strains, whereas 10 T9SS-related cargo proteins were identified only in the OMVs of specific strains.

To verify the presence of the T9SS-related OMV cargo proteins Kgp, RgpA/B and PPAD in the investigated OMVs, LDS-PAGE and Western-blotting analyses were performed using specific antibodies (Fig. 2). In this analysis, the OMV association of the non-T9SS-related outer membrane protein Omp41 was assessed for control. This demonstrated the presence of Kgp, RgpA/B and Omp41 in all OMVs. As expected, PPAD was detected only in OMVs of the wild-type ATCC 33277 and W83 strains as well as the clinical isolates, but not in OMVs of the ATCC 33277 and W83 strains from which the PPAD gene had been deleted. Furthermore, as previously shown (Gabarrini et al., 2018a), OMVs from the strains with the PPAD sorting type I presented a PPAD-specific band of 75–85 kDa, which was strongly reduced in OMVs from the clinical isolates with the PPAD sorting type II. In contrast, OMVs from the clinical isolates presented two PPAD-specific bands at 47 kDa and 37 kDa, which are characteristic for the PPAD sorting type II (Gabarrini et al., 2018a).

An interesting observation was that in all OMVs analyzed by proteomics, we identified PorQ PorU, PorV and PorZ, which are the four components that make up the so-called T9SS attachment complex (Veith et al., 2017). For the ATCC 33277 strain and its PPAD-deficient derivative the attachment complex components were the only T9SS components identified (Table 3). Similarly, a previous study showed that the T9SS attachment complex was enriched in OMVs of the W50 strain when compared to the whole bacterial cells (Veith et al., 2018). The apparent absence of other T9SS components from the OMVs of ATCC 33277 is consistent with previous findings, indicating that the attachment complex is not tightly associated with other T9SS components (Gorasia et al., 2020b; Veith et al., 2018). Nonetheless, our findings demonstrate that additional T9SS components can be packaged into the OMVs of *P. gingivalis*. In particular, all known components of the T9SS system were detected in OMVs of strain W83, except those components that are localized in the cytoplasm and/or the inner membrane (Table 3). Interestingly, OMVs of the PPAD-deficient W83 mutant did not seem to contain the T9SS components PorE, PorF, PorG and PorP. Both PorG and PorP were previously detected in high amounts in OMVs of *P. gingivalis* W50 when this strain was abundantly supplied with heme (Veith et al., 2018), similar to our present findings for OMVs of the PPAD-proficient W83 strain. In this context it is noteworthy that PorF was previously reported as being required for the biogenesis of gingipains (Saiki et al., 2010). Of note, our present Western blotting analyses show that RgpA and RgpB are present in comparable amounts in OMVs from PPAD-proficient and -deficient W83 bacteria (Fig. 2G). This is consistent with our recent observation that PPAD is required for the optimal arginine-specific activity of these two gingipains (Xu et al., 2026), which suggests that the reduced activity of RgpA/B in absence of PPAD could be associated with the absence of PorE, PorF, PorG and PorP.

**Table 3:** T9SS system components identified in OMVs of the investigated *P. gingivalis* strains.

^a^T9SS component	Gene locus tag ATCC 33277	Gene locus tag W83	^b^Predicted localization	^c^Identification in OMVs					
				W83	W83 ΔPPAD	ATCC 33277	ATCC ΔPPAD	505759	512915
PorX	PGN_1019	PG0928	CYT/ IM	—	—	—	—	—	—
PorY	PGN_2001	PG0052		—	—	—	—	—	—
PorL	PGN_1675	PG0289		—	—	—	—	—	—
PorM	PGN_1674	PG0290		—	—	—	—	—	—
PorN	PGN_1673	PG0291	Periplasm	**×**	**×**	—	—	**×**	**×**
PorK	PGN_1676	PG0288		**×**	**×**	—	—	**×**	**×**
PorW	PGN_1877	PG1947		**×**	**×**	—	—	—	—
OmpA family protein (PorE)	PGN_1296	PG1058		**×**	—	—	—	—	—
Plug*	PGN_0144	PG2092		**×**	**×**	—	—	—	—
Sov	PGN_0832	PG0809	OM/Surface	**×**	**×**	—	—	—	**×**
PorQ**^#,^**^†^	PGN_0645	PG0602		**×**	**×**	**×**	**×**	**×**	**×**
PorP	PGN_1677	PG0287		**×**	—	—	—	—	**×**
PorT	PGN_0778	PG0751		**×**	**×**	—	—	**×**	**×**
PorV**^#,^**^†^	PGN_0023	PG0027		**×**	**×**	**×**	**×**	**×**	**×**
β-barrel protein (PorG)	PGN_0297	PG0189		**×**	—	—	—	—	—
Ton-B dependent receptor (PorF)	PGN_1437	PG0534		**×**	—	—	—	—	—
Omp17	PGN_0300	PG0192		**×**	**×**	—	—	—	**×**
PorU**^#,^**^†^	PGN_0022	PG0026		**×**	**×**	**×**	**×**	**×**	**×**
PorZ**^#,^**^†^	PGN_0509	PG1604		**×**	**×**	**×**	**×**	**×**	**×**

^a^T9SS component: *, marks proteins added to the list in this study compared to Lasica *et al*. (2017); **#**, marks proteins that are detected in OMVs of all strains; **†**, marks the components of the T9SS attachment complex. **^b^Predicted localization:** Based on Lasica *et al*. (2017): CYT, cytoplasmic; IM, inner membrane; OM, outer membrane. **^c^Identification in OMVs:** identified proteins are marked with **×**. Proteins not detected in the respective OMVs were marked with —.

Regarding the other T9SS components detectable in OMVs, we also identified the Sov translocon in OMVs of the W83 strain, of its PPAD-deficient derivative, and of clinical isolate 512915. Sov is the largest outer membrane β-barrel protein ever described (Gorasia et al., 2020a). Interestingly, the protein Plug, which was identified as a T9SS component that binds to the periplasmic opening of Sov (McBride, 2019), was also detectable in OMVs of the PPAD-proficient and -deficient W83 strains. The possible mechanisms by which PPAD may influence the sorting of T9SS system components to the outer membrane of *P. gingivalis* cells and to OMVs have been described in previous studies (Lasica et al., 2017; Gabarrini et al., 2018c). Altogether, the presently identified differential sorting of T9SS components into OMVs are suggestive of a differential interaction between these components in the different investigated strains. Our findings also hint at a direct or indirect role of citrullination in the interactions between the T9SS components, which would explain the apparent absence of the T9SS components PorE, PorF, PorG and PorP from the OMVs of the PPAD-deficient W83 strain.

#### 3.1.4 Citrullination of OMV-associated proteins

Since PPAD-catalyzed citrullination is an important theme in the pathogenesis of *P. gingivalis*, we inspected the identified OMV proteins for possible citrullination sites. As expected, no citrullination sites were detected in OMV proteins of the two PPAD-deficient mutants. In contrast, a citrullination site was detected in the Q7MT41 (PG2150) protein in OMVs derived from the W83 strain (**Supplementary File S3**), and this modification was subsequently confirmed by manual validation. Notably, the same citrullination site was also identified by orthogroup analysis in the corresponding proteins from the clinical isolates 505759 and 512915 (orthogroup OG0000329). An additional citrullination site was identified in the Q7MUK3 protein in W83-derived OMVs.The low number of identified citrullination sites possibly reflects a limitation of the applied MS-based methodology as the mass difference due to citrullination is merely 1 Da. This problem was addressed in a publication by Larsen et al. (2020) in which a new separation system together with the use of an in-house built program specifically designed to identify and validate citrullination sites was proposed to yield a much greater number of identified citrullination sites in OMVs of *P. gingivalis.* Unfortunately, we were unable to implement this program in our present study.

### 3.2 Definition of core and variant OMV proteomes of investigated *P. gingivalis* strains

#### 3.2.1 Relatively minor differences in OMV-proteomes of PPAD-proficient or -deficient *P. gingivalis* reference strains suggest correlations between OMVs, PPAD and heme acquisition

To assess the extent by which PPAD influences the composition of the OMV proteome, we performed pair-wise comparisons of the OMV proteomes of isogenic PPAD-proficient and PPAD-deficient W83 and ATCC 33277 strains. As shown in Fig. 3, comparable numbers of proteins were identified in OMVs of the W83 type strain and its PPAD-deficient derivative (Fig. 3A), and the same was true for OMVs of the ATCC 33277 strain and its PPAD-deficient derivative (Fig. 3B). Because these wild-type and mutant pairs share an identical genome annotation, this analysis was performed as a direct protein-level comparison rather than using orthogroups. As expected, the PPAD protein was exclusively identified in OMVs of the two wild-type strains and absent from OMVs of the mutant strains. In general, OMVs of PPAD-proficient and -deficient strain pairs were found to have about 82 to 85% of the identified proteins in common. In particular, the OMVs of the W83 and W83ΔPPAD strains shared 182 out of 215 identified proteins, while OMVs of the ATCC 33277 and ATCCΔPPAD strains shared 95 out of 116 identified proteins. Only between 15 and 18% of the identified proteins were uniquely identified in either the wild-type strains or the respective PPAD-deficient mutants. As expected from this high overlap (Fig. 3), only few differences in the predicted localization of OMV-associated proteins of PPAD-proficient and -deficient strains were detected (Fig. 1). Thus, it seems that a lack of PPAD may affect the overall sorting of proteins into the OMVs of *P. gingivalis* only to a relatively minor extent.

**Fig. 3:**
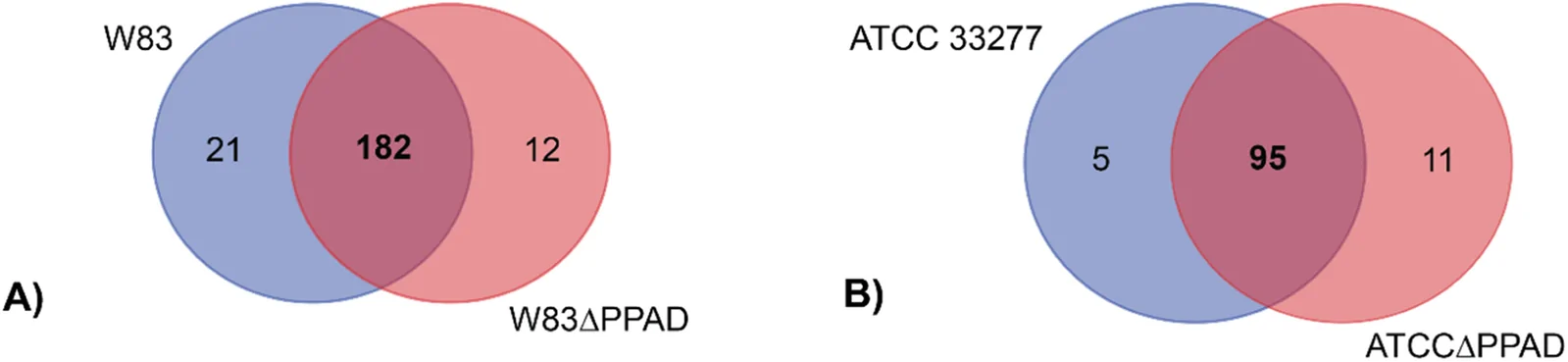
**Venn diagrams comparing the numbers of identified proteins in OMVs from PPAD-proficient or PPAD-deficient *P. gingivalis* strains. (A)** Numbers of identified OMV proteins in the wild-type strain W83 versus the W83ΔPPAD mutant. **(B)** Numbers of identified OMV proteins in the wild-type strain ATCC 33277 versus the ATCCΔPPAD mutant.

All the proteins uniquely identified by the pairwise comparisons of the OMV proteomes of the W83 and W83ΔPPAD strain are presented in Table 4. In total, 21 and 12 proteins were uniquely identified in the OMV-proteomes of the W83 and W83ΔPPAD strains, respectively. For instance, the OMVs of the W83 strain, but not those of the W83ΔPPAD strain, uniquely contain a sialidase (PG0352), which cleaves sialic acid from host glycoproteins*.* In particular, this sialidase was shown to be important for *P. gingivalis* survival under stressful environmental conditions, biofilm formation, capsule biosynthesis, protection against killing by complement, and regulation of the activity of gingipains and other virulence factors (Heath et al., 2016; Frey et al., 2019; Xu et al., 2017; Aruni et al., 2011; Yu et al., 2021). Furthermore, as mentioned above, the OMVs of the PPAD-deficient W83 strain lack four components of the T9SS secretion system, namely PorE, PorF, PorG and PorP. On the contrary, in absence of PPAD, several proteins from the inner membrane and cytoplasm were uniquely identified in OMVs of the W83ΔPPAD strain. For instance, these include a conserved uncharacterized cytoplasmic hemerythrin domain-containing protein (PG0686) that is upregulated when *P. gingivalis* invades epithelial and endothelial cells (Bélanger et al., 2006). In addition, two fimbrial components, Mfa2 and Mfa3, were uniquely identified in OMVs of the W83ΔPPAD strain.

**Table 4:** Proteins uniquely identified in OMVs of the *P. gingivalis* W83 PPAD-proficient or PPAD-deficient strains.

Unique proteins	Protein	aUniprot Code	Gene locus tag	^b^Predicted localization
Detected in of W83 OMVs, but not in W83ΔPPAD OMVs	Uncharacterized protein	Q7MW22*	PG0840	CYT
	DUF58 domain-containing protein	Q7MUE8	PG1580	
	α-l-fucosidase, putative	Q7MTR2	PG1884	
	META domain-containing protein	Q7MWC2	PG0706	IM_LP
	Uncharacterized protein related to GLPGLI family protein (A0A2D2NBN7)	Q7MWZ0	PG0438	IM_TM
	Uncharacterized protein related to PF11644 family protein (T2N847)	Q7MUU3	PG1388	
	β-mannosidase, putative	Q7MXW7	PG0032	Periplasm
	TPR domain protein	Q7MXA8	PG0300	
	Sialidase	Q7MX62	PG0352	
	Uncharacterized protein	Q7MWV7	PG0479	
	SPOR domain-containing protein	Q7MWI6	PG0624	
	α−1,2-mannosidase family protein	F5HDD2	PG1712	
	Thioredoxin domain-containing protein	Q7MT23	PG2175	
	β-barrel protein (PorG)	Q7MXJ5	PG0189	OM
	Collagen-binding protein	Q7MWY8	PG0441	
	Ton-B dependent receptor (PorF)	Q7MWR1	PG0534	
	Uncharacterized protein	Q7MTI1	PG1978	
	Por secretion system protein (PorP)	Q7MXB8	PG0287	
	OmpA family protein (PorE)	F5HF80	PG1058	OM_LP
	Peptidylarginine deiminase (PPAD)	Q9RQJ2	PG1424	Secreted T9SS
	DUF5683 domain-containing protein	Q7MXN2	PG0140	unknown
Detected in W83ΔPPAD OMVs, but not W83 OMVs	Uncharacterized protein related to Biopolymer transporter ExbD (A0A134DR34)	Q7MW65	PG0780	CYT
	Elongation factor Tu	F5HDQ9	PG0387	
	Hemerythrin domain-containing protein	Q7MWD6	PG0686	
	HlyD family secretion protein	F5HI17	PG0093	IM_TM
	Uncharacterized protein, related to PF04402 family protein (T2NAT9)	Q7MX59	PG0356	
	YngK protein	Q7MWV9	PG0476	IM_LP
	Efflux transporter, MFP component, RND family	Q7MWQ7	PG0539	
	Uncharacterized protein	Q7MUA1	PG1635	Periplasm
	Uncharacterized protein related to Right-handed beta helix region domain protein (A0A0A2F7P5)	Q7MXF7	PG0236	
	LptD – LPS transport system	Q7MUS9	PG1405	OM
	Minor fimbrium anchoring subunit Mfa2	Q7MXK2	PG0179	OM_LP
	Minor fimbrium tip subunit Mfa3	Q7MXK1	PG0180	

a **Uniprot Code:** *Variable genome. **^b^Predicted localization:** CYT, Cytoplasmic; IM_LP, inner membrane lipoprotein; IM_TM, transmembrane inner membrane; OM, outer membrane; OM_LP, outer membrane lipoprotein; T9SS, type IX secretion system.

The uniquely identified proteins in OMVs of the ATCC 33277 and ATCCΔPPAD strains are presented in Table 5. In total, 5 and 11 proteins were uniquely identified in the OMV-proteomes of the ATCC 33277 and ATCCΔPPAD strains, respectively, by pair-wise comparison. The gingipain-sensitive surface ligand (0748), which was previously shown to activate the nuclear factor (NF)-κB in a reporter cell-line (Haruyama et al., 2009), was only detected in OMVs of the ATCCΔPPAD strain. This is consistent with the notion that PPAD is required for optimal gingipain activity (Xu et al., 2026). Likewise, the fimbriation-related protein FimX (PGN_0178) was exclusively detected in OMVs lacking PPAD, and the same was true for the periplasmic dipeptidyl peptidase IV (PGN_1469), which cleaves proteins into dipeptides at X-Ala and X-Pro sites for nutrient acquisition (Kumagai et al., 2005). Interestingly, besides having protease activity, the dipeptidyl aminopeptidase IV also promotes the adhesion of *P. gingivalis* to human fibronectin and destruction of connective tissue (Kumagai et al., 2005; Ohara-Nemoto et al., 2018).

**Table 5:** Proteins uniquely identified in OMVs of the *P. gingivalis* ATCC 33277 PPAD-proficient or PPAD-deficient strains.

Unique proteins	Protein	Uniprot Code	Gene locus tag	aPredicted localization
Detected in ATCC 33277 OMVs, but not in ATCC ΔPPAD OMVs	Putative DNA methylase	B2RGW0	PGN_0086	CYT
	Lipoprotein	B2RHL9	PGN_0345	IM_LP
	C69-family unassigned peptidase	B2RJS7	PGN_1103	Periplasm
	Uncharacterized protein related to the Phosphate-selective porin O and P (A0A0K2J3V0)	B2RJ34	PGN_0860	OM_LP
	Peptidylarginine deiminase (PPAD)	B2RJ72	PGN_0898	Secreted T9SS
Detected in ATCC ΔPPAD OMVs, but not in ATCC 33277 OMVs	Gingipain-sensitive ligand	B2RIS2	PGN_0748	CYT
	Glycine dehydrogenase (aminomethyl-transferring)	B2RJR8	PGN_1094	
	Dipeptidyl aminopeptidase IV	B2RKU3	PGN_1469	Periplasm
	Uncharacterized protein	B2RHJ7	PGN_0323	
	Uncharacterized protein. Related to: YtxH-like protein (T2N7N4)	B2RMB8	PGN_1995	
	OMP β barrel 2 domain-containing protein	B2RMF3	PGN_2030	OM
	Probable immunoreactive 23 kDa antigen	B2RI06	PGN_0482	
	Putative alkaline protease AprF	B2RMG4	PGN_2041	
	FimX	B2RH52	PGN_0178	
	PEGA domain-containing protein	B2RIT2	PGN_0758	OM_LP
	TSPc domain-containing protein	B2RHZ4	PGN_0470	

a **Uniprot Code:** *Variable genome. **^b^Predicted localization:** CYT, cytoplasmic; IM_LP, inner membrane lipoprotein; OM, outer membrane; OM_LP, outer membrane lipoprotein; T9SS, type IX secretion system.

Lastly, the protein levels in OMVs of PPAD-proficient and PPAD-deficient *P. gingivalis* strains were analyzed based on MaxQuant log₂‑transformed LFQ intensities (**Supplementary File S4**). This highlighted in the first place that T9SS-related OMV cargo proteins are highly enriched components of the OMV proteome. Specifically, 20 of the 30 predicted T9SS cargo proteins (67%) in the W83 strain clustered within the top abundance quartile of the OMV proteome **(Supplementary File S4, sheet 1).** In OMVs from the ATCC 33277 strain, 10 of the 25 predicted T9SS proteins (40%) were identified within the top abundance quartile of the OMV proteome. These results verify previous observations, where T9SS-dependently secreted virulence factors were found to be enriched in the OMVs of *P. gingivalis* (Veith et al., 2014). Furthermore, upon pairwise comparisons of the T9SS-related cargo proteins in W83ΔPPAD OMVs versus wild‑type W83 OMVs, we detected a 1.73-fold increase of periodontain (PG1427), which is a putative cysteine protease with hemagglutinin activity (Madden et al., 1995; Nelson et al., 1999), (Table 6). In contrast, the T9SS component PorT was reduced by 1.63‑fold in W83ΔPPAD OMVs (Table 6). PorT is part of the T9SS in *P. gingivalis,* which is essential for the degradation of hemoglobin by gingipains and heme-capture (Lasica et al., 2017). Decreased levels of PorT affect the enzymes required by *P. gingivalis* to harvest heme (Lasica et al., 2017). Furthermore, PG0709 (also known as FkpA), which is a putative peptidyl-prolyl cis-trans isomerase, was significantly decreased by 1.9-fold. This protein could potentially act as a catalyst of protein folding in the context of heme-chaperoning systems. It has been documented FkpA regulates protein fate in *P. gingivalis* and in other Gram-negative bacteria it modulates rapid folding, translocation and maturation of outer membrane proteins (Yuan et al., 2007; Taguchi et al., 2016). In addition to T9SS‑related OMV cargo proteins, significant differences were also observed for non‑T9SS-related proteins. In OMVs of the ATCCΔPPAD strain, a 1.79-fold increase of the cytoplasmic moonlighting protein GAPDH (PGN_0173) was detected (Table 6). The presence of GAPDH in OMVs of various bacterial species has been previously reported, and it is known to promote OMV adhesion to several host cell surfaces or even membrane fusion (Whitworth et al., 2015). Interestingly, the previous study by Veith et al. (2018) showed that cytoplasmic proteins, like GAPDH, were significantly more abundant in OMVs of the W83 strain when the bacteria were cultured in the presence of excess of heme than under heme-depleted conditions. This suggests that cytoplasmic proteins such as GAPDH could potentially function in a relevant coping mechanism for OMV-mediated heme uptake in PPAD-deficient *P. gingivalis*. Notably, compared to OMVs of the wild-type W83 strain, the TonB-dependent outer membrane heme receptor HmuR (PG1552) showed the strongest (2.85-fold) reduction in abundance in OMVs of the PPAD-deficient W83 strain (Table 6). The HmuR protein and its cognate lipoprotein, the hemopore-like protein HmuY, are known to capture heme at the bacterial cell surface for uptake into the periplasm (Simpson et al., 2000; Olczak et al., 2024). Moreover, moonlighting GAPDH has been implicated in the binding of heme that can be sequestered by hemophore-like proteins such as HmuY (Ślęzak et al., 2020). Together, these observations are indicative of a possible correlation between OMVs, PPAD and heme acquisition.

**Table 6:** Proteins with increased or decreased abundance in OMVs of PPAD-deficient *P. gingivalis* strains compared to the respective PPAD-proficient wild-type strains.

OMV comparison	Protein name	UniProt	Gene locus tag	Predicted Locali-zation^a^	Fold change	Direction of fold change
W83ΔPPAD versus W83	OMP β-barrel domain protein	Q7MXP8	PG0123	OM	1.51	**↓**Decreased
	GLPGLI-family related protein	Q7MTP2	PG1908	CYT	1.53	
	OMP β-barrel 2 protein	Q7MTV9	PG1823	OM	1.53	
	Uncharacterized protein	Q7MT69	PG2112	CYT	1.57	
	OMP β-barrel domain protein	Q7MTC8	PG2041	OM	1.57	
	Uncharacterized protein	Q7MWJ7	PG0613	Periplasm	1.64	
	Uncharacterized protein	Q7MU17	PG1757	OM_LP	1.65	
	PorT	F5HG90	PG0751	OM	1.67	
	Uncharacterized protein, related to: DUF5020 domain-containing protein (CBG53_09805)	Q7MVU6	PG0937	Periplasm	1.71	
	Rhodanese-domain protein	Q7MU51	PG1713	OM_LP	1.82	
	Peptidyl-prolyl cis-trans isomerase FkpA	Q7MWC0	PG0709	Periplasm	1.9	
	TonB-dependent receptor HmuR	Q7MUG9	PG1552	OM	2.85	
	Outer membrane protein 40	Q9S3R8	PG0694	OM	1.66	**↑**Increased
	Periodontain (PrtT)	Q7MUR6	PG1427	Secreted (T9SS)	1.73	
	MotA/TolQ/ExbB family protein	Q7MW63	PG0782	IM_TM	5.0	
ATCCΔPPAD versus ATCC 33277	Uncharacterized protein, related to: tRNA(Glu)-specific nuclease WapA (A0A1R4DVU2)	B2RLU2	PGN_1818	OM	1.79	**↓**Decreased
	Glyceraldehyde-3-phosphate dehydrogenase (GAPDH)	B2RH47	PGN_0173	Extra-cellular, no SP	1.79	**↑**Increased

a **Predicted localization:** CYT, Cytoplasmic; IM_TM, inner membrane-transmembrane; OM, outer membrane; OM_LP, outer membrane lipoprotein; T9SS, type IX secretion system; no SP, no signal peptide.

The protein with the highest (5-fold) increase in OMVs of the W83ΔPPAD strain compared to the W83 strain, was the inner membrane protein Q7MW63 (PG0782), which was also previously detected in OMVs of the W83 strain (Veith et al., 2014). This protein belongs to the MotA/TolQ/ExbB family of proton channels that appear to be involved in protein translocation across membranes. Furthermore, ExbB and ExbD, together with outer membrane receptors, like HmuR, HusB, or IhtA, are thought to form a complex that facilitates proton-motive force-driven translocation of heme and iron across the cytoplasmic membrane (Śmiga, and Olczak, 2025). Additionally, the Omp40 protein (PG0694) was found to be enriched in OMVs of the W83ΔPPAD strain (Table 6). Omp40 is predicted to form 8-stranded β-barrels in its N-terminal domains. Importantly, since its C-terminal domain is known to bind peptidoglycan, it seems likely that Omp40 is important for the structural integrity of the outer membrane (Veith et al., 2021). In contrast, in the OMVs of the W83 strain, three β-barrel domain-containing outer membrane proteins (i.e. PG1823, PG0123 and PG2041) were found in higher quantities when compared to OMVs of the W83ΔPPAD strain. PG1823 is an 8-stranded β-barrel protein that was predicted to help maintain OMV structure (Gui et al., 2016). Furthermore, PG0123 is possibly involved in virulence since, similar to the T9SS-related OMV cargo proteins, it has a low number of homologues in other bacteria belonging to the *Bacteroidales* (Veith et al., 2021).

#### 3.2.2 OMV-proteomes of PPAD sorting type I and sorting type II strains suggest links between reduced OM(V)-bound PPAD and alterations in fimbrial composition or assembly

The overlaps and differences in the numbers of OMV-associated orthogroups of the W83 and ATCC 33277 wild-type strains and the clinical isolates 505759 and 512915 are shown in Fig. 4A. This comparison shows that a total of 79 core OMV orthogroups were identified in all strains, which we regard as the OMV core proteome as detailed in **Supplementary File S2, sheet 2**. However, since the overlap of identified OMV orthogroups of the ATCC 33277 strain was smallest with the identified OMV orthogroups in the other strains, we also determined the overlap of common OMV orthogroups from only the W83, 512915 and 505759 strains. Indeed, this revealed a greater overlap in the identified OMV-associated proteins of these three strains, with 128 common orthogroups (Fig. 4B; **Supplementary File S2, sheet 2**). This shows that the OMVs of the two clinical isolates bear the highest resemblance to OMVs of the W83 strain in terms of their protein content. Representative proteins present in the core OMV proteome of *P. gingivalis* identified in our study include those involved in adhesion (HagA, PG1837; FimA, PG2132), proteolysis (HtrA, PG0593), heme acquisition and nutrient uptake (HmuY, PG1551; FetB, PG0669; RagA, PG0185; and RagB, PG0186), as well as the virulence factor PPAD (PG1424), and the outer membrane proteins Omp28 (PG2173), Omp40 (PG0182), and Omp41 (PG2164).

**Fig. 4:**
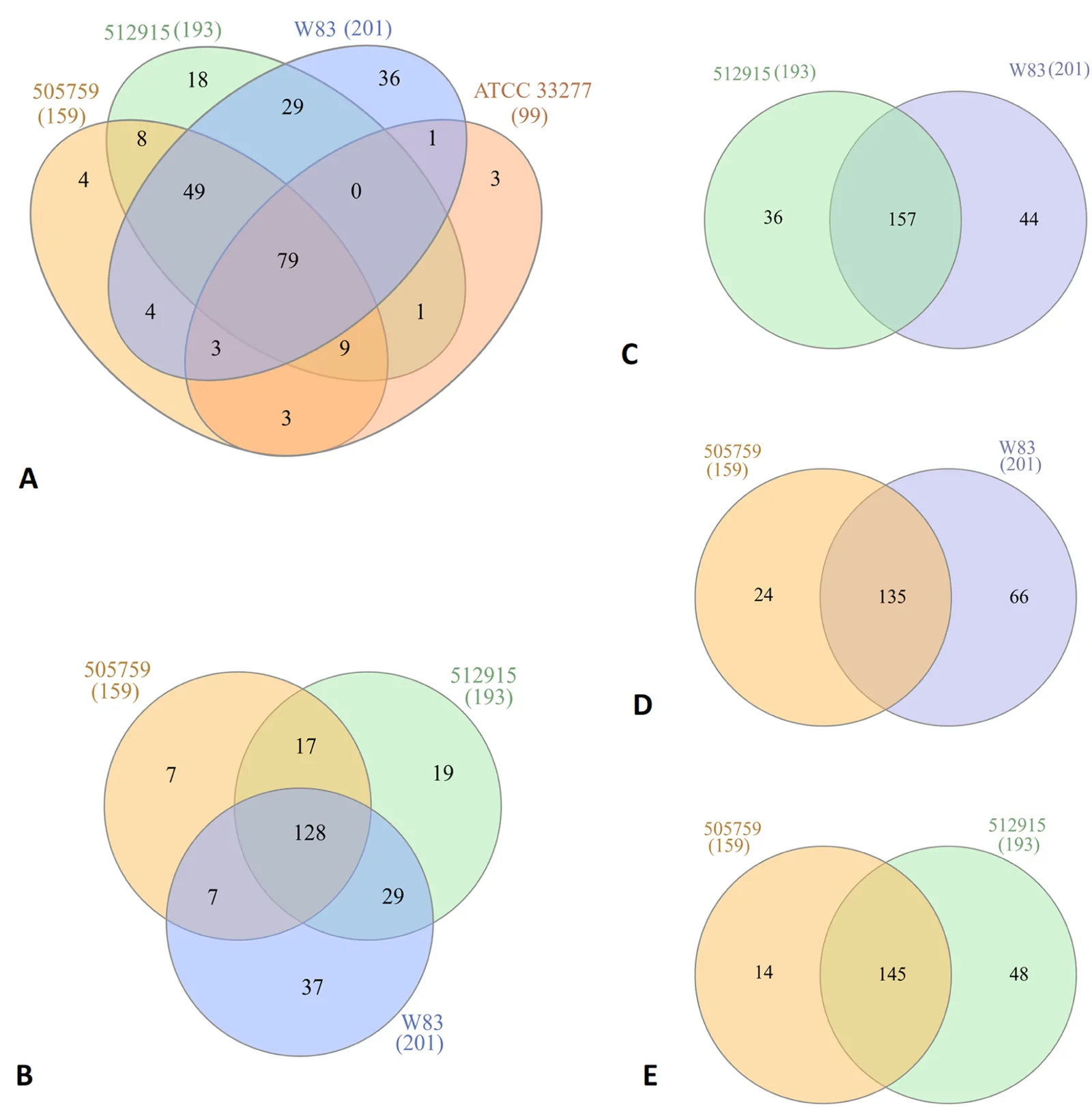
**Venn diagrams showing the numbers of common and unique orthogroups identified in OMVs of the investigated strains.** The total number of orthogroups is indicated in parentheses under each strain name. **(A)** Comparison of the numbers of OMV-associated orthogroups in the *P. gingivalis* type strains W83 and ATCC 33277 (both PPAD sorting type I) and the clinical (PPAD sorting type II) strains 505759 and 512915. **(B)** Numbers of identified OMV-associated orthogroups in the type strain W83 and the clinical isolates 512915 and 505759. **(C)** Numbers of identified OMV-associated orthogroups in the type strain W83 versus the clinical isolate 512915. **(D)** Numbers of identified OMV-associated orthogroups in the wild-type strain W83 versus the clinical isolate 505759. **(E)** Numbers of identified OMV-associated orthogroups in the clinical isolate 505759 versus the clinical isolate 512915.

In addition, a pair-wise comparative analysis was performed for the W83, 512915 and 505759 strains. A total number of 157, 135 and 145 shared orthogroups were, respectively, detected in OMVs of strains W83 and 512915, strains W83 and 505759, and strains 512915 and 505759 (Fig. 4, C-E; **Supplementary File S2, sheet 4**). Furthermore, compared to the OMV-associated orthogroups of the W83 strain, a subset of orthogroups appeared to be strain-specific, with 36 OMV-associated proteins unique for strain 512915 and 24 OMV-associated proteins unique for strain 505,759. Interestingly, 77 of the 79 core proteins (97.5%) were identified in at least one of the published W50 OMV proteomes, while 67 proteins were shared among all three datasets. Targeted analysis of the 79 OMV core proteins identified in this study in comparison with other publications (Veith et al. 2014; Veith et al. 2018) revealed that 77 of the 79 core proteins (97.5%) present in the OMVs from the reference strains W83, ATCC 33277 and clinical isolates 505759 and 512915, were also identified in at least one of the published W50 OMV proteomes, while 67 proteins were identified across all three datasets (**Supplementary File S2, sheet 3; Supplementary Figure S3).** The two proteins that were exclusively found in our 79-protein OMV core proteome were F5H9J5 (PG0538) and Q7MU49 (PG1715). Interestingly, proteins of the PG0538–PG0541 efflux system and PG1715, which is related to TonB-dependent receptors (TBDRs), have in common that they modulate heme-uptake and iron homeostasis in *P. gingivalis* (Simpson et al., 2000; Veith et al. 2018; Olczak et al., 2024; Cierpisz et al., 2026).

To identify general functions of the 79 core OMV-associated orthogroups of *P. gingivalis* W83, ATCC 33277, 505759 and 512915 (Fig. 4A), a REVIGO analysis was performed. As graphically represented in Fig. 5, the respective proteins were assigned to 11 biological pathways. Amongst the 11 biological pathways represented in all investigated OMVs are pathogenesis, proteolysis, cell adhesion, redox homeostasis and oxidation–reduction processes, which are all related to the pathogenic lifestyle of *P. gingivalis*. The latter is consistent with the view that the release of OMVs from the bacterial outer membrane is a major mechanism of *P. gingivalis* for targeting virulence factors to cells and tissues of its human host (Gabarrini et al., 2020). The other six biological pathways invariably represented in OMVs of strains W83, ATCC 33277, 505759 and 512915 relate to the acquisition and metabolism of nutrients.

**Fig. 5:**
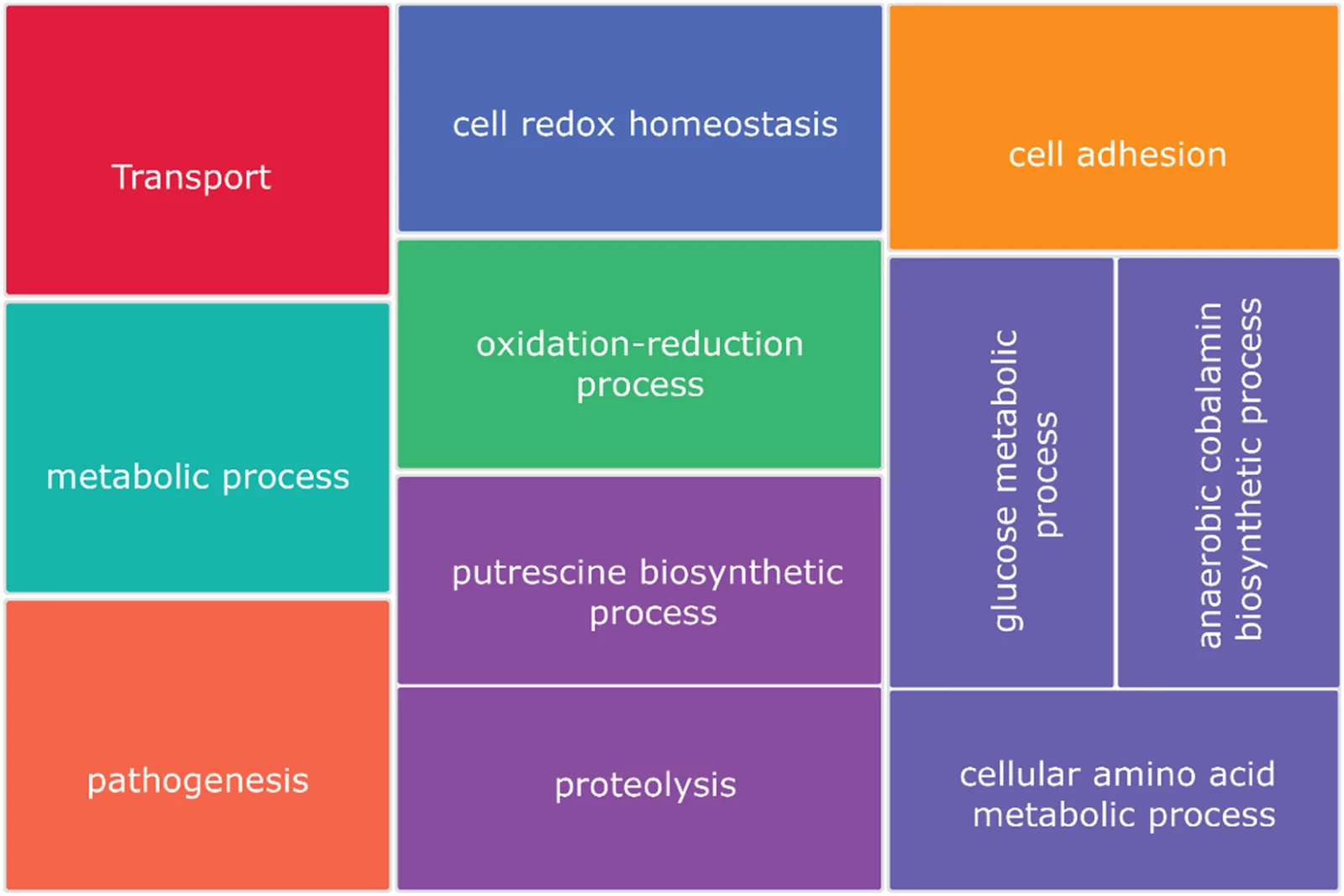
REVIGO tree map of biological pathways represented by orthogroups identified in OMVs of the *P. gingivalis* strains W83, ATCC 33277, 505759 and 512915.

REVIGO analyses of the uniquely identified orthogroups in OMVs of the W83 reference strain versus the clinical strains 512915 and 505759 (Fig. 4C,D), revealed that proteins related to carbohydrate metabolic processes, fucose metabolism, oxidation–reduction processes and peptidoglycan catabolism were consistently represented in OMVs of the W83 strain in contrast to OMVs of the clinical isolates (Fig. 6). Furthermore, upon pairwise comparison of the functions of uniquely identified proteins in the OMV-proteome of the two clinical isolates against OMVs from the W83 strain, various different functions in biological pathways were identified. These include siderophore and protein transport, signal peptide processing, electron transport, redox processes, extracellular polysaccharide biosynthesis and proteolysis (Fig. 6). Several of the uniquely identified orthogroups of the clinical strains 505759 and 512915 were also shared with OMVs of the ATCC 33277 strain (marked with * in **Supplementary File S2, sheet 4),** including proteins involved in cell-cell adhesion and pathogenesis, such as fimbriae-related proteins.

**Fig. 6:**
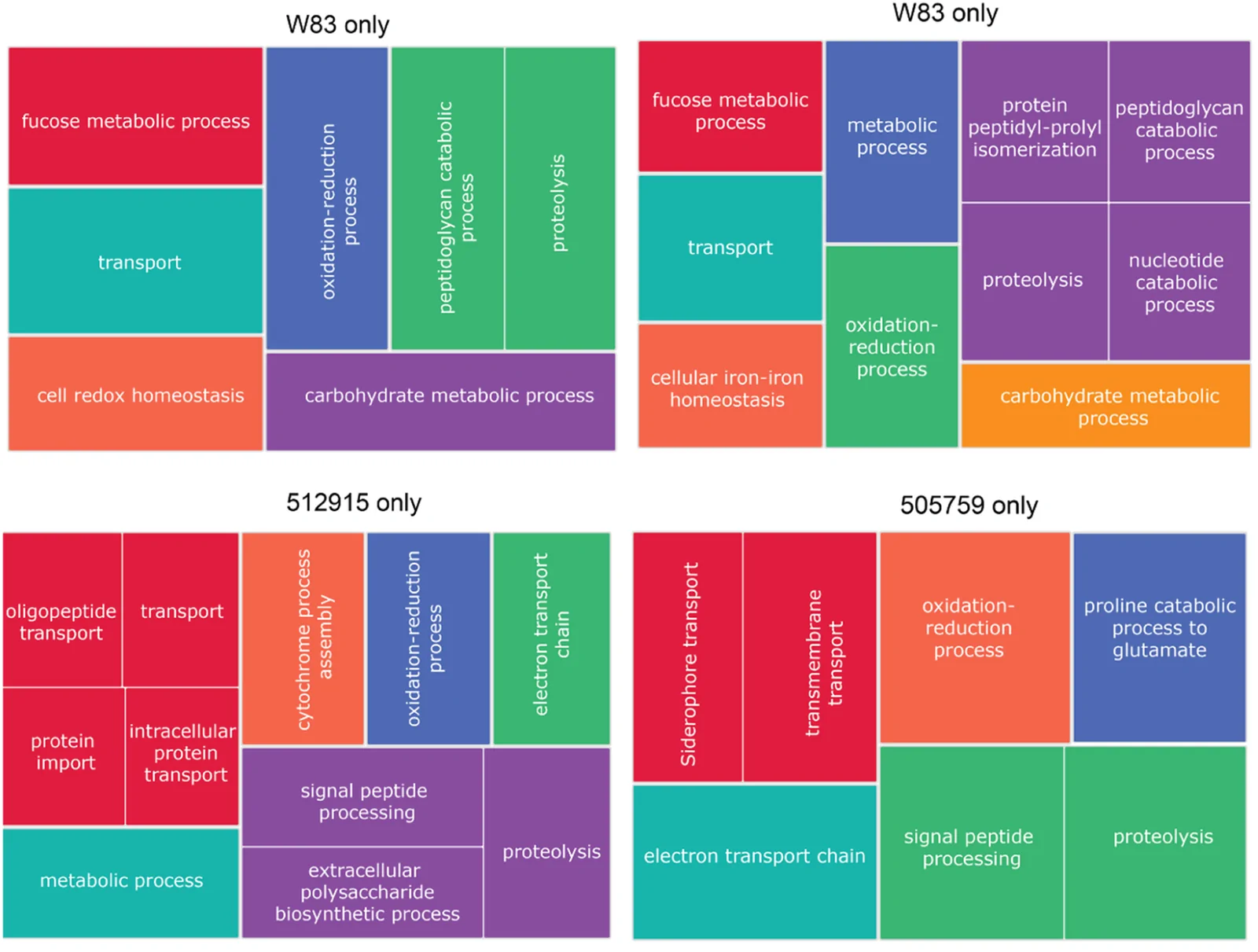
**REVIGO treemaps of biological pathways represented by uniquely identified orthogroups in OMVs of the clinical *P. gingivalis* isolates 512915 or 505759 compared to orthogroups uniquely identified in OMVs of the W83 type strain.** The REVIGO treemaps on the left show the uniquely identified biological pathways related to orthogroups in the OMVs of strain W83 versus clinical isolate 512915 (corresponding to Fig. 4C), and REVIGO treemaps on the right show the uniquely identified biological pathways related to orthogroups in the OMVs of strain W83 versus clinical isolate 505759 (corresponding to Fig. 4D).

To further explore the potential roles of PPAD, we analyzed whether proteins uniquely present in OMVs of PPAD-deficient type strains (Table 3, Table 4, Table 5) would be present in OMVs from the clinical isolates with PPAD sorting type II, which contain substantially reduced amounts of OM(V)-bound PPAD (Fig. 2F). The results of this comparison are presented in Table 7. Among the proteins uniquely detectable in OMVs from PPAD-deficient type strains and also in type II isolates, we identified several proteins associated with fimbrial structure and localization, such as the minor fimbrial anchoring subunit Mfa2 and the minor fimbrial tip subunit Mfa3. In addition, the FimX protein was detected in the clinical isolate 512915. This protein encoded in the *fim* gene cluster is a transcriptional regulator controlling the expression of the *fimA* gene (Nagano et al., 2012). Altogether, these observations suggest potential links between reduced OM(V)-bound PPAD and alterations in fimbrial composition or assembly, which could tentatively impact the adhesion capacity of OMVs, subsequent host-pathogen interactions and biological functions related to pathogenicity. So far, reports from Vermilyea et al. (2019) and Xu et al. (2026) have demonstrated that PPAD mutants from *P. gingivalis* strain 381 and W83 present alterations in their extracellular matrix production and biofilm formation capacities, respectively. Also, the absence of PPAD from *P. gingivalis* was previously shown to impact the adhesion of OMVs to, and internalization by neutrophils and macrophages, as well as the induction of bacterial aggregation (Stobernack et al., 2018; du Teil Espina et al., 2022a, 2022b; Xu et al., 2026). Further studies are needed to test whether the absence of PPAD causes an impactful change in the fimbrial composition and the bacterial microbe-microbe and microbe-host interactions of *P. gingivalis*.

**Table 7:** Proteins detected only in OMVs of PPAD-deficient *P. gingivalis* type strains and PPAD sorting type II clinical isolates.

Protein name	Uniquely detected in OMVs of ATCC ΔPPAD vs ATCC 33277	Uniquely detected in OMVs of W83ΔPPAD vs W83	Clinical PPAD sorting type II isolate(s) in which the protein is detected	aPredicted localization
Uncharacterized protein	-	Q7MW65	505759 & 512915	CYT
Uncharacterized protein	-	Q7MX59	512915	IM_TM
Putative cation efflux system protein	-	Q7MWQ7	505759 & 512915	IM_LP
Uncharacterized protein	-	Q7MUA1	512915	Periplasm
Uncharacterized protein	-	Q7MXF7	505759 & 512915	Periplasm
FimX	B2RH52		512915	OM
Minor fimbrium anchoring subunit Mfa2	-	Q7MXK2	505759 & 512915	OM_LP
Minor fimbrium tip subunit Mfa3	-	Q7MXK1	505759 & 512915	OM_LP

a **Predicted localization:** CYT, Cytoplasmic; IM_TM, inner membrane-transmembrane; IM-LP, inner membrane lipoprotein; OM, outer membrane; OM_LP, outer membrane lipoprotein.

### 3.3 Interaction of *P. gingivalis* OMVs with human red blood cells

#### 3.3.1 OMVs from *P. gingivalis* trigger hemagglutination of human RBCs with strain-specific PPAD-dependency

At least six OMV-associated proteins were identified in our present proteomics analyses that can be linked to interactions with RBCs, especially hemagglutination (Table 2**, Supplementary File S2, sheet 1**). Among these, the most noticeable one was the hemagglutinin protein HagA (PG1837), which is a well-known mediator of hemagglutination and hemoglobin binding to facilitate heme acquisition (Shi et al., 1999). Importantly, HagA was detected in OMVs from all investigated *P. gingivalis* strains. Furthermore, Kgp and RgpA were detected in all investigated OMVs. These two gingipains possess related hemagglutinin/adhesion domains and were previously reported to be synergistically engaged in heme supply (Okamoto et al., 1996; Nakayama et al., 1998; Śmiga et al., 2023). Likewise, we identified in the OMVs from all investigated strains the cysteine protease/hemagglutinin PrtT (PG1427), which has a minor role in hemagglutination and is homologous to the streptococcal pyrogenic exotoxin B (Madden et al., 1995; Nelson et al., 1999). Lastly, two putative hemagglutinins (PG0076 and PG0411) were detected in all investigated OMVs, but their specific biological function and actual contributions to hemagglutination remain to be elucidated.

To assess whether OMVs of *P. gingivalis* can induce hemagglutination, we performed *in vitro* hemagglutination assays with human RBCs. The results presented in Fig. 7A demonstrate, for the first time, that OMVs from *P. gingivalis* are indeed capable of inducing high-level hemagglutination of hRBCs with HAt values ranging from 0.078–0.625 µg/mL of total OMV protein content. Remarkably, the HAt values determined for the *P. gingivalis* OMVs were, in fact, ∼400- to 50-fold lower than the concentration needed to induce the same hemagglutinating effect with the lectin PHA-P from kidney beans, which served as a positive control for hemagglutination (Fig. 7B). In particular, the OMVs from the W83, W83ΔPPAD, and ATCC 33277 strains and those from the clinical isolate 505759 presented the highest hemagglutinating effect, as shown by their HAt values of 0.078 µg/mL. OMVs from the ATCCΔPPAD strain and clinical isolate 512915 showed HAt values of 0.313 µg/mL and 0.625 µg/mL, respectively, indicating a lower potency for hemagglutination.

**Fig. 7:**
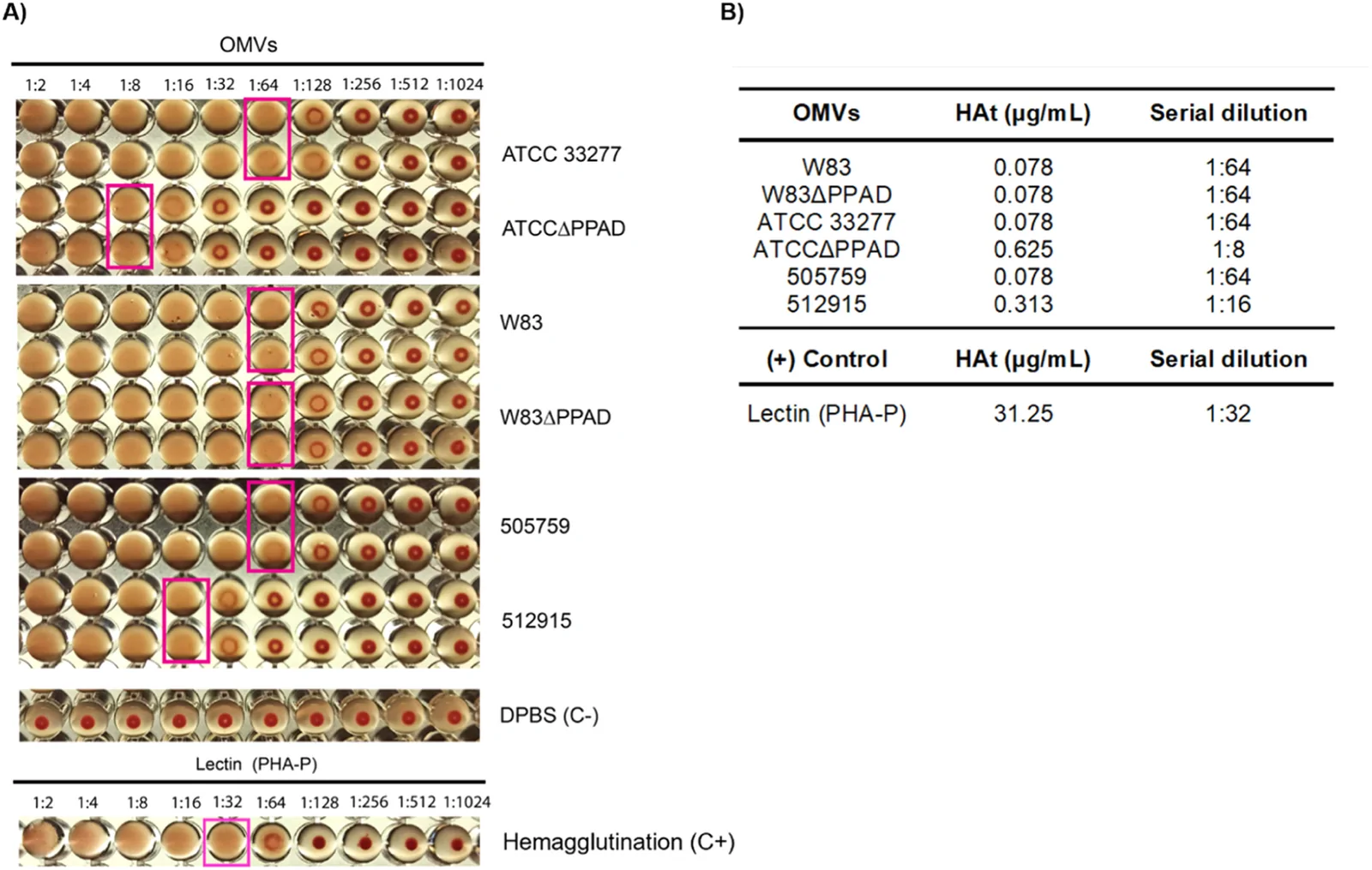
**Hemagglutination of human RBCs caused by *P. gingivalis* OMVs. (A)** Picture of a U-shaped 96-well microplate showing hemagglutination of hRBCs induced by OMVs from the six investigated *P. gingivalis* strains or the positive (C+) control lectin (PHA-P). The initial undiluted concentration of the OMV stocks was 5 µg/mL (total protein content) and the initial undiluted concentration of the lectin stock was 1000 µg/mL. Pink squares indicate the Hemagglutination-titer (Hat). For negative control (C-), DPBS was added to the hRBCs. **(B)** HAt concentrations (µg/mL) determined by double serial dilutions.

The HAt value measured for OMVs of the ATCCΔPPAD strain was ∼8-fold higher than the HAt of OMVs of the parental ATCC 33277 strain. In contrast, this PPAD-dependent effect on hemagglutination was not observed for OMVs of the W83 strain. This suggests that particular proteins, which are apparently absent from OMVs of the PPAD-deficient ATCC 33277 strain, but not from OMVs of the PPAD-deficient W83 strain, contribute to hemagglutination. One candidate protein that fulfils this criterion is the uncharacterized protein PGN_0860 (Table 5), which is related to the ‘Porin_O_P family of anion-selective 16-stranded porins’. Structural homology modeling using Phyre2 (Veith et al., 2021; Powell et al., 2025) and visualization with the EzMol webserver (Reynolds et al., 2018), indicates similarity of this porin to well-characterized outer membrane porins, such as OmpF from *Escherichia coli*, OprP from *Pseudomonas aeruginosa* and OmpU of *Vibrio cholerae* (**Supplementary Figure S4**). Notably, OmpU of *V. cholerae* has been implicated in the hemagglutination of rabbit and human RBCs (Chakrabarti et al., 1996; Pathania et al., 2018). Together, these findings indicate that the absence of PGN_0860 could contribute to hemagglutination mediated by OMVs from ATCC 33,277 strain. Since the level of the PGN_0860 homologue PG1382 was not affected in PPAD-deficient OMVs of the W83 strain, these observations imply that PPAD is a context-dependent factor for hemagglutination of hRBCs mediated by OMVs from *P. gingivalis.* However, here it is important to bear in mind that the non-detection of a protein by MS does not necessarily mean that this protein is actually completely absent from the analyzed samples (Table 5, Table 6). Thus, it is conceivable that, in addition, other proteins that are absent or present in reduced amounts in OMVs from the PPAD-deficient ATCC 33277 strain contribute to hemagglutination. Moreover, our recent studies have shown that RgpA and RgpB present significantly reduced proteolytic activity in the absence of PPAD, whereas the levels of these proteins were comparable in OMVs from PPAD-deficient and -proficient bacteria (Xu et al., 2026). Since especially RgpA is less abundant in OMVs of the ATCC 33277 strain than in OMVs of the W83 strain (Fig. 2E), a lowered activity of RgpA may have a higher impact on hemagglutination provoked by OMVs of the ATCC 33277 strain. Thus, the reduced hemagglutination presented by OMVs from the PPAD-deficient ATCC 33277 strain could be due to a combination of reduced RgpA activity, the absence of PGN_0860 and other factors.

The second noteworthy observation related to OMV-triggered hemagglutination was that OMVs from the W83 and ATCC 33277 type strains and the clinical isolate 505759 presented comparably high hemagglutinating effects, whereas OMVs from the clinical isolate 512915 presented a much lower potency for hemagglutination. This implies that one or more of the 54 proteins detected in OMVs from the W83, ATCC 33277 and 505759 strains, but absent from the 512915 isolate (Fig. 4A), could be responsible for this difference in hemagglutination. However, it is also conceivable that differences in the abundance or activity of proteins with known roles in hemagglutination, or proteins not yet known to be involved in hemagglutination, could be responsible for the relatively lower potency for hemagglutination presented by OMVs from the clinical *P. gingivalis* 512915 isolate. Unfortunately, based on the presently available data, it is not possible to pinpoint such proteins. Instead, this will require a combination of genetic approaches and proteomics analyses that allow absolute protein quantification per OMV.

Lastly, it was interesting to observe that a PG1881-related protein (B2RLT2, Q7MTR3) is present amongst the 79 core OMV proteins of the strains analyzed in our study (**Supplementary File S2, sheet 2)**. In a previous study, it was proposed that binding and aggregation of sheep RBCs could be mediated by the OMV-associated fimbriae-like lipoprotein PG1881 of *P. gingivalis* (Rothenberger et al., 2024). Of note, the PG1881-related protein is one of the top 25 decreased proteins in OMVs from the ATCC∆PPAD strain in comparison to OMVs from the parental ATCC 33277 strain (**Supplementary File S4, sheet 2)**. In contrast, this protein was one of the top 25 significantly increased proteins in OMVs from the W83∆PPAD strain in comparison to OMVs from the W83 parental strain (**Supplementary File S4, sheet 1)**. Altogether, our results seem to suggest that the PG1881-related protein could perhaps also be relevant for OMV-mediated hemagglutination of hBRCs, and that the reduction of OMV-mediated hemagglutination activity towards hRBCs in the absence of PPAD from OMVs of the ATCC 33277 strain could be correlated to the decreased abundance of the PG1881-related protein in the respective OMVs (Fig. 7). On the contrary, absence of PPAD from the W83 strain led to an increase of the PG1881-related protein in OMVs without a noticeable change in OMV-mediated hemagglutination activity towards hRBCs (Fig. 7). Altogether, these findings suggest that *P. gingivalis* OMV-mediated hemagglutination of hRBCs may involve the PG1881-related protein in a strain-specific manner.

#### 3.3.2 Hemolytic activity of *P. gingivalis* bacteria and OMVs

Our present analyses of the *P. gingivalis* OMV proteomes identified not only proteins implicated in hemagglutination, but also several proteins related to hemolysis. In particular, the gingipain RgpB (PG0506) has been reported to degrade RBC membrane proteins, such as the glycophorin A, resulting in increased RBC sensitivity for lysis (Li et al., 2010; Li and Collyer, 2011; Smalley and Olczak, 2017). On the other hand, RgpA (PG2024) is known to oxidize hemoglobin into methemoglobin. The methemoglobin is subsequently degraded by Kgp (PG1844) to release free heme that is captured by the bacteria as a nutritional iron source (Smalley et al., 2011; Śmiga et al., 2023). Furthermore, the OMV-borne cysteine protease periodontain (PrtT; PG1427) has been implicated in the maturation of Kgp and Rgp (Kadowaki et al., 2000; Nelson et al., 1999). Other OMV-associated proteases that could potentially help *P. gingivalis* to break down host proteins and participate in heme uptake, are the putative zinc-carboxypeptidase (PG0232) and a 35-kDa hemin binding protein with putative thioredoxin activity (PG0616). It was previously demonstrated that starvation caused by the absence of fully active heme uptake systems in a *P. gingivalis* ∆*hmuYR* mutant strain led to upregulation of, for instance, the PG0232 gene (Śmiga et al., 2024). Also, in another study, the recombinant 40 kDa HBP35 protein exhibited thioredoxin and hemin-binding activities (Shoji et al., 2010). Of note, hemin-binding proteins, such as FetB (also known as IhtB) are putative bacterial metal chelatases, which are key modulators in iron/heme-acquisition and porphyrin metabolism (Śmiga, and Olczak, 2025; Phonok et al., 2026). Hence, these previous studies provide further support for potential roles of OMV-borne proteins in hemin acquisition by *P. gingivalis*, but their potential effects on hRBCs are yet unknown.

To assess whether OMVs from *P. gingivalis* can elicit hRBC lysis and, if so, whether their hemolytic activity would be influenced by PPAD, or by the PPAD sorting type, we performed *in vitro* hemolysis assays with hRBCs. The results show that, indeed, the OMVs of all the six investigated *P. gingivalis* strains caused RBC lysis in a dose-dependent manner (Fig. 8). Importantly, all investigated OMVs were also hemolytic according to the ASTM F-756-00 guidelines (ASTM, 2020), since they were capable of causing >5% hRBC lysis. Furthermore, the observed effects were strain-specific, as was verified by a linear regression analyses according to the least squares method, which showed a strong fit for the hemolysis data (R^2^ = 0.985; p-value ≤ 0.0001), with both the strain type and the OMV concentration being significant factors for hemolysis (p < 0.0001; **Supplementary Figures S5 and S6**). A Tukey post hoc analysis detected no significant differences in the high hemolytic activity presented by OMVs from the PPAD-proficient or PPAD-deficient ATCC 33277 and W83 type strains (**Supplementary Table S2**). However, this analysis showed that (i) the hemolytic activities of OMVs from the clinical isolates 512915 and 505759 were significantly lower than those from the two type strains, and that (ii) they also differed significantly from each other, with OMVs from clinical isolate 512915 showing intermediate hemolytic activity and OMVs from isolate 505759 showing low hemolytic activity (**Supplementary Table S2**). Altogether, the results show that OMVs from the investigated PPAD sorting type I strains were significantly more hemolytic than those produced by clinical isolates with the PPAD sorting type II.

**Fig. 8:**
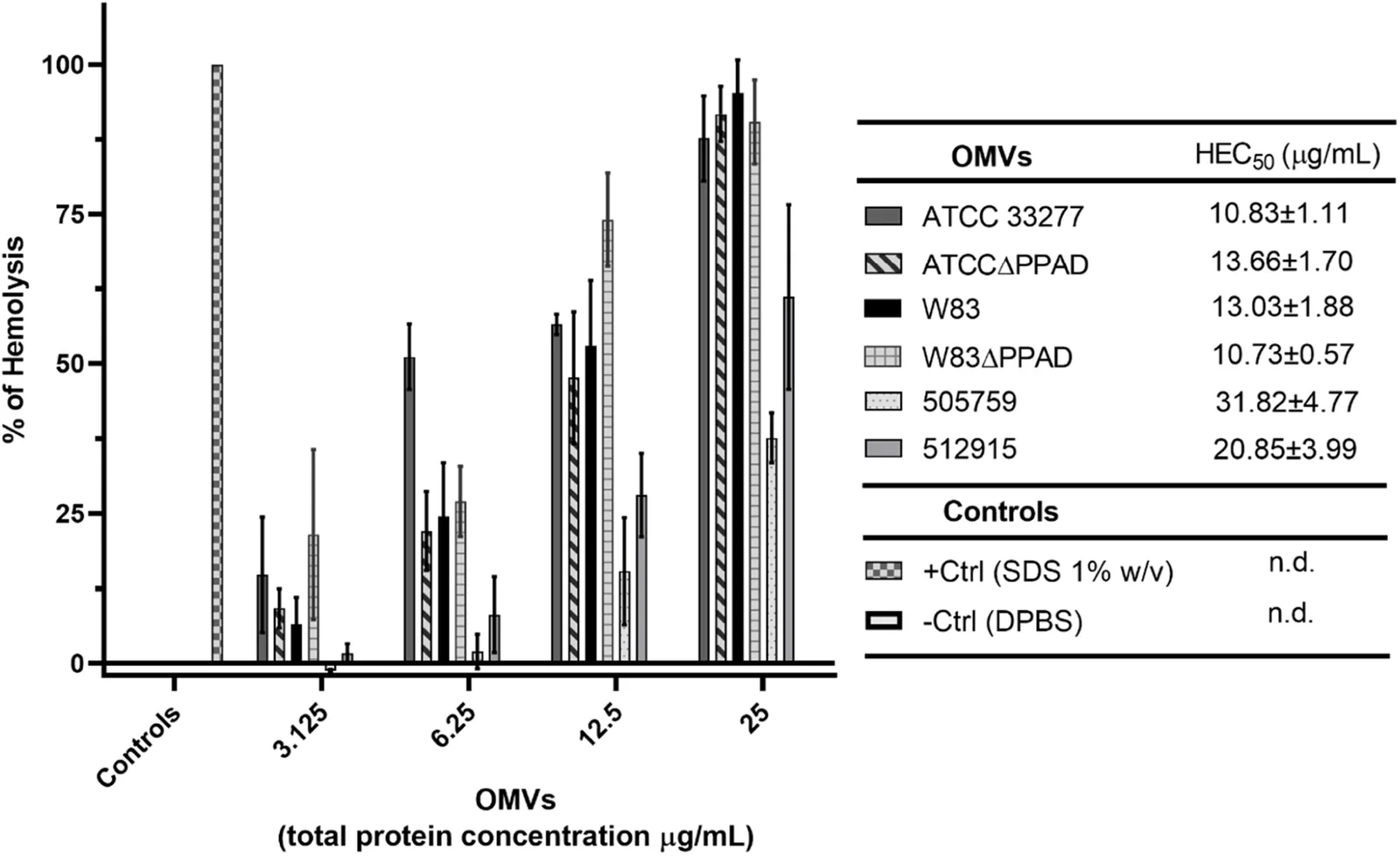
**Hemolytic activity of *P. gingivalis* OMVs.** Hemolytic activity of OMVs from the PPAD-proficient or PPAD-deficient *P. gingivalis* W83 and ATCC 33277 strains, and from the clinical isolates 505759 and 512915, was assayed at four serially diluted concentrations (25–3.125 µg/mL of total protein content). The results are plotted as the arithmetic mean value of the hemolysis (%) ± standard deviation (marked by error bars). Each condition was tested in two biologically independent experiments with two technical replicates (n = 48). The HEC_50_ values (µg/mL) are shown next to each type of OMVs. These values represent the concentration of *P. gingivalis* OMVs (based on total protein content) capable of inducing hemolysis in 50% of the hRBCs after 48 h of incubation at 37 °C. n.d., indicates that values were **n**ot **d**etermined since the controls were performed with 1% SDS and DPBS.

The observation that OMVs from PPAD-deficient and -proficient type strains display equally strong hemolytic activities shows that PPAD is dispensable for OMV-mediated hemolysis. This is at least partially different from what we observed for OMV-mediated hemagglutination, and it implies that any differences in the levels of the OMV-associated major gingipains Kgp, RgpA and RgpB, or their enzymatic activity, do not impact the hemolytic potency of the respective OMVs (Fig. 2E,G). Furthermore, the relatively lower hemolytic activities observed for OMVs from clinical PPAD sorting type II isolates suggest that strain-specific differences in OMV composition, rather than PPAD alone, determine the extent of OMV-mediated hemolysis. These differences are quite substantial as OMVs from clinical isolate 512915 differed in 50 identified proteins from the OMVs of the two type strains, whereas OMVs from clinical isolate 505759 differed in 88 proteins from the OMVs of the three other strains investigated in this study (Fig. 4A). In addition, also here, it is important to consider that OMV normalization based on total protein content, as performed in our present study, does neither capture absolute differences in the amounts of protein molecules per OMV, nor potential differences in the vesicle sizes, net particle charge, or protein localization within OMVs. Such factors may affect the accessibility and interaction of OMV-associated proteins with the hRBCs, thereby influencing the outcome of the hemolysis assay.

## 4 Conclusions

In the present study, we have analyzed the proteomes of OMVs produced by two PPAD-proficient or PPAD-deficient *P. gingivalis* type strains with the PPAD sorting type I, as well as two clinical *P. gingivalis* isolates with the PPAD sorting type II. The total numbers of proteins identified in OMVs from different strains ranged between 101 and 203 proteins that predominantly represent outer membrane and periplasmic proteins. Based on the respective protein identifications, we conclude that OMVs released by different *P. gingivalis* isolates present isolate-specific proteomic signatures, which implies that these OMVs may have different pathogenic effects in the human body. Nonetheless, amongst the identified proteins, 79 common OMV orthogroups were distinguished. Furthermore, together the investigated OMVs presented a total of 23 T9SS-related cargo proteins, including the major virulence factors of *P. gingivalis* and constituents of the T9SS machinery. The PorQ, PorU, PorV and PorZ proteins that make up the so-called T9SS attachment complex (Veith et al., 2017) were identified in all investigated OMVs. Interestingly, OMVs of the W83 type strain contained all known periplasmic and OM-bound T9SS machinery components, but these were not identified in OMVs of the PPAD-deficient W83 strain. This finding suggests a potential link between PPAD and its citrullinating activity in assembly of the T9SS machinery, and it focuses attention on another intriguing observation, namely that PPAD-deficiency correlated with differential effects on the protein content of OMVs from the W83 and ATCC 33277 strains. Our search for common features of OMVs from PPAD-deficient strains and OMVs from the PPAD sorting type II strains that contain reduced amounts of PPAD suggests potential links between reduced OMV-bound PPAD and alterations in fimbrial composition or assembly. This could impact the adhesion capacity of OMVs, subsequent host-pathogen interactions and biological functions related to pathogenicity, albeit that clinical isolates with the PPAD sorting type II are still pathogenic since they were derived from patients with periodontitis (Gabarrini et al., 2018a).

Lastly, the results of our proteome analyses focus attention on potentially important roles of OMVs in iron acquisition and the involvement of PPAD in the respective processes. In particular, HmuR (Q7MUG9) which is essential for iron acquisition was found to be present at significantly reduced levels in OMVs of the PPAD-deficient strain W83 strain. Moreover, we identified at least six OMV-borne proteins involved in hemagglutination. Accordingly, we demonstrated for the first time that OMVs from *P. gingivalis*, invariably have a very high potency for hemagglutination of human RBCs, albeit that we observed differences in the hemagglutinating activity of OMVs from different strains. Here it was noteworthy that the hemagglutinating activity of OMVs from the PPAD-deficient type strain ATCC 33277 was significantly reduced compared to its PPAD-proficient counterpart, which could be associated to the absence of the porin-like PGN_0860 protein from these OMVs, or to a previously demonstrated reduced activity of the gingipain RgpA in absence of PPAD (Xu et al., 2026). The investigated OMVs of *P. gingivalis* also displayed significant hemolytic activity towards hRBCs, albeit at concentrations that were about 80- to 300-fold higher than the concentrations needed for hemagglutination. This suggests that OMV-mediated hemagglutination could be a major mechanism employed by *P. gingivalis* for interaction with hRBCs. In line with this notion is the fact that we did not identify the major hemolysin (PG1875; Smalley and Olczak, 2017) in the investigated OMVs. Altogether, our present observations disclose different interactions of *P. gingivalis* OMVs with human RBCs related to heme and iron acquisition, and they also make it plausible that attachment to hRBCs could represent a mechanism for OMV dissemination throughout the human body via the bloodstream. Further *in vivo* and epidemiological studies are needed to test this intriguing concept.

## Ethics declaration

Written informed consent to take part in the study and to publish the article has been obtained from all participants or their legal representatives. The privacy rights of participants have been observed.

This study included organ or tissue donors. This study includes human biological material and consent was obtained by donors, or their next of kin or legal representatives, for use in this study and for publication of the article. The samples used in this research were not sourced from executed prisoners or prisoners of conscience.

This study was performed in compliance with relevant laws, regulatory frameworks and guidelines where the research took place. This study was conducted in accordance with Declaration of Helsinki guidelines. This study was approved by the Medical ethics committee of the University Medical Center Groningen (UMCG). (Approval No. Metc2012-375)

## Data requirements

All MS data have been deposited to the ProteomeXchange Consortium via the PRIDE partner repository (Vizcaíno, J. A., Csordas, A., Del-Toro, N., Dianes, J. A., Griss, J., Lavidas, I., et al.,2016. 2016-update of the PRIDE database and its related tools. Nucleic Acids Res. 44, D447–D456. doi: 10.1093/nar/gkv1145) with the dataset identifier **PXD067239**.

## Declaration of generative AI in scientific writing

The authors declare that this manuscript was written without the use of generative AI and AI-assisted technologies.

## Funding sources

R.d.C.F.V. acknowledges the financial support from CONACyT-Mexico PhD scholarship [Grant No 773,955], the Graduate School of Medical Sciences, Groningen University and the European Institute of Innovation and Technology (EIT) Healthy ageing PhD program.

## CRediT authorship contribution statement

**Marines du Teil Espina:** Conceptualization, Methodology, Investigation, Formal analysis, Writing – original draft, Writing – review & editing, Visualization. **Rosario del Carmen Flores-Vallejo:** Conceptualization, Methodology, Investigation, Formal analysis, Data curation, Visualization, Writing – original draft, Writing – review & editing. **Claudia Hirschfeld:** Investigation, Formal analysis, Writing – review & editing. **Stefano Grasso:** Investigation, Formal analysis, Data curation, Writing – review & editing. **Pim Rozendal:** Investigation, Formal analysis. **Anke Trautwein-Schult:** Investigation, Formal analysis, Writing – review & editing. **Dörte Becher:** Investigation, Formal analysis, Resources. **Giorgio Gabarrini:** Formal analysis, Supervision, Writing – review & editing. **Jan Maarten van Dijl:** Conceptualization, Resources, Funding acquisition, Supervision, Writing – original draft, Writing – review & editing, Project administration.

## Declaration of competing interest

The authors declare the following financial interests/personal relationships which may be considered as potential competing interests:

Rosario del Carmen Flores-Vallejo reports financial support was provided by CONACyT-Mexico. Rosario del Carmen Flores-Vallejo reports financial support was provided by Graduate School of Medical Sciences of the University of Groningen. Rosario del Carmen Flores-Vallejo reports financial support was provided by European Institute of Technology (EIT) Healthy ageing PhD program. If there are other authors, they declare that they have no known competing financial interests or personal relationships that could have appeared to influence the work reported in this paper.
