# Supplementary material for: Interaction of outer membrane vesicles of the oral pathogen Porphyromonas gingivalis with human red blood cells disclosed by proteome analyses
Source: Curr Res Microb Sci. 2026 Oct 30;11:100674. doi: 10.1016/j.crmicr.2026.100674 (PMC13744359; doi:10.1016/j.crmicr.2026.100674)
Supplement: Supplementary file 1 [file mmc1.docx]

**Supplementary Materials**

**Interaction of outer membrane vesicles of the oral pathogen *Porphyromonas gingivalis* with human red blood cells disclosed by proteome analyses**

Marines du Teil Espina^a,b,#^, Rosario del Carmen Flores-Vallejo^a,c,#^, Claudia Hirschfeld^d,e,π^, Stefano Grasso^a,f,π^, Pim Rozendal^a^, Anke Trautwein-Schult^e^, Dörte Becher^e^, Giorgio Gabarrini^a,g†^, and Jan Maarten van Dijl^a†*^

^a^University of Groningen, Department of Medical Microbiology, University Medical Center Groningen, 9713 GZ, Groningen, The Netherlands.

^b^Wageningen Bioveterinary Research, 8221 RA Lelystad, The Netherlands

^c^European Institute of Innovation and Technology (EIT), Healthy Ageing PhD School. Campus University Medical Center Groningen, University of Groningen, 9713 GZ, Groningen, The Netherlands.

^d^University of Greifswald, Department of Microbial Physiology and Molecular Biology, Institute of Microbiology, 17489, Greifswald, Germany.

^e^University of Greifswald, Department of Microbial Proteomics, Institute of Microbiology, 17489, Greifswald, Germany.

^f^Lesaffre International, 101 Rue de Menin, Marcq-en-Barœul 59700, Marquette-lez-Lille, France.

^g^ImmunoIVD, Nacka Strand, 13152, Stockholm, Sweden.

^#^These authors contributed equally and share first authorship

^π^These authors contributed equally and share second authorship

^†^These authors contributed equally and share last authorship

*Corresponding author. E-mail address: j.m.van.dijl01@umcg.nl

| **Content** | **Page** |
| --- | --- |
| **Supplementary Figure S1.** OMV isolation- and purification-related images | **3** |
| **Supplementary Figure S2.** Representative images of *P. gingivalis* OMVs recorded via Nanoparticle Tracking Analysis (NTA) | **4** |
| **Supplementary Figure S3.** Comparison of the 79 core proteins of *P. gingivalis* OMVs identified in this study with OMV-associated proteins identified in previous studies | **5** |
| **Supplementary Figure S4.** *In silico-*generated structural model of the PGN_0860 protein of *P. gingivalis* | **6** |
| **Supplementary Figure S5.** Fitness analysis of the Linear Regression model for OMV-induced hemolysis based on the Least Squares Method (LR-LSM) | **7** |
| **Supplementary Figure S6.** Pareto´s plot showing differences between the independent experimental factors that contribute the most to explain the variance in the percentage of hemolysis | **8** |
| **Supplementary Table S1.** Physicochemical properties of purified *P. gingivalis* OMVs | **9** |
| **Supplementary Table S2.** Results from the Tukey´s post hoc test to evaluate differences in hemolytic activities of OMVs from the different investigated *P. gingivalis* strains | **10** |

**Supplementary Figure S1. OMV isolation- and purification-related images. (A)** Growth curves of the analyzed *P. gingivalis* strains. Highlighted in red is the time window (22-24 h) at which OMVs were harvested. **(B)** Picture of an ultracentrifuge tube showing the characteristic band that corresponds to OMVs in an iodixanol density gradient created by ultracentrifugation. **(C)** Coomassie staining of LDS-PAGE gel loaded with different fractions obtained from the iodixanol gradient in panel C. **(D)** Western-blotting to visualize the presence of the Omp41 protein, which shows that OMVs were concentrated in the fractions corresponding to 20% and 30% iodixanol.

**Supplementary Figure S2. Representative images of *P. gingivalis* OMVs recorded via Nanoparticle Tracking Analysis (NTA)**. OMVs from **(A)** *P. gingivalis* ATCC 33277; **(B)** *P. gingivalis* ATCC∆PPAD; **(C)** *P. gingivalis* 505759; and **(D)** *P. gingivalis* 512915. The respective videos in NTA mode (Supplementary Videos S1-S4) were recorded at 11 positions across the measurement chamber (n=33).

**Supplementary Figure S3. Comparison of the 79 core proteins of *P. gingivalis* OMVs identified in this study with OMV-associated proteins identified in previous studies**. The Venn diagram depicts the numbers of shared and unique orthogroups between the 79 core proteins of OMVs from *P. gingivalis* strains W83, ATCC 33277, 505759 and 512915, and the OMV proteome from *P. gingivalis* W50 (Veith et al., 2014; Veith et al., 2018).

**Figure S4. *In silico-*generated structural model of the PGN_0860 protein of *P. gingivalis***. The PGN_0860 protein (B2RJ34) belongs to the ‘Porin_O_P family of anion-selective 16-stranded porins’, showing similarity to the OmpF, OprP and OmpU proteins of *Escherichia coli*, *Pseudomonas aeruginosa* and *Vibrio cholerae*, respectively. OmpU of *Vibrio cholerae* was previously implicated in hemagglutination of rabbit and human erythrocytes. The structure of PGN_0860 was modelled via structural homology using the Phyre-2 algorithm and visualized with the EZMOL webserver. Amino acidic sequence length: 306 residues. Phyre output results: Confidence (100.0%); Coverage (88%); Model dimensions (Å): X:50.148; Y:43.080 Z:62.218.

**Supplementary Figure S5. Fitness analysis of the Linear Regression model for OMV-induced hemolysis based on the Least Squares Method (LR-LSM).** **(A)** Predicted versus observed values of % of Hemolysis (n= 48; R^2^= 0.985; Root Mean Square Error (RMSE)= 6.721; p-value≤0.0001). **(B)** Histogram and Boxplot describing the distribution of the residuals.

**Supplementary Figure S6. Pareto´s plot showing differences between the independent experimental factors that contribute the most to explain the variance in the percentage of hemolysis.** The *P. gingivalis* strain from which OMVs where isolated and the OMVs concentration contribute significantly (p<0.0001) to the observed effect on the response variable, i.e. the % of hemolysis.

**Supplementary Table S1. Physicochemical properties of purified *P. gingivalis* OMVs**

| *P. gingivalis* OMVs | Size  (nm)^a^ | Zeta potential (mV)^a^ | Concentration  (particles/mL)^b^ |
| --- | --- | --- | --- |
| ATCC | 100.85±2.70 | -47.07±3.12 | 4.50 ±1.59 × 10^12^ |
| ATCC∆PPAD | 106.73±3.42 | -43.12±1.43 | 4.72 ± 0.97 ×10^12^ |
| 512915 | 120.37±10.74 | -38.58±13.42 | 1.33 ± 0.48×10^12^ |
| 505759 | 120.67±12.03 | -40.45±5.66 | 3.70 ± 0.77×10^11^ |

^a^ Size and zeta potential of OMVs (100 µg/mL) obtained after 1:1x10^5^ to 1:3x10^5^ dilution in DPBS (pH 7.0); ^b^ Concentration of OMVs (particles/mL) present in a stock normalized to 100 µg/mL of total protein content in each sample. All measurements were obtained from 3 biological replicates by Nanoparticle Tracking Analysis (NTA). Videos in NTA mode (Supplementary Videos S1-S4) were recorded at 11 positions across the measurement chamber (n=33). Data is presented as the mean value ± standard deviation.

**Supplementary Table S2.** Results from the Tukey´s post hoc test to evaluate differences in hemolytic activities of OMVs from the different investigated *P. gingivalis* strains

| Type of Bacterial strain | Levels^a^ | | | Root Mean Square  (RMS) |
| --- | --- | --- | --- | --- |
| W83ΔPPAD | A |  |  | 53.262 |
| ATCC 33277 | A |  |  | 52.532 |
| W83 | A |  |  | 44.786 |
| ATCC 33277ΔPPAD | A |  |  | 42.681 |
| 512915 |  | B |  | 24.773 |
| 505759 |  |  | C | 13.444 |

^a^Levels not connected by the same letter are significantly different. A refers to the highest hemolytic activity and C to the lowest hemolytic activity.
